# Loss of *Brca1* and *Trp53* in adult mouse mammary ductal epithelium results in development of hormone receptor-positive or hormone receptor-negative tumors, depending on inactivation of Rb family proteins

**DOI:** 10.1186/s13058-022-01566-4

**Published:** 2022-11-04

**Authors:** Ludmila Szabova, Melanie B. Gordon, Lucy Lu, Nathan Pate, Laura Bassel, Anthony J. Iacovelli, Baktiar Karim, Philip J. Homan, Deborah B. Householder, Theresa M. Guerin, Sandra Burkett, Amanda M. Day, Wendi Custer, Zoe Weaver Ohler

**Affiliations:** 1 Center for Advanced Preclinical Research, Frederick National Laboratory for Cancer Research, National Cancer Institute, Frederick, MD USA; 2 Center for Advanced Preclinical Research, Center for Cancer Research, National Cancer Institute, National Institutes of Health, Frederick, MD USA; 3 Present Address: Sanofi,Global Discovery Pathology, Translational In Vivo Models Platform, Framingham, MA USA; 4 Present Address: Genentech, Inc., South San Francisco, CA USA; 5 Present Address: Molecular Histopathology Laboratory, Frederick National Laboratory for Cancer Research, National Cancer Institute, Frederick, MD USA; 6 CCR Collaborative Bioinformatics Resource, Center for Cancer Research, National Cancer Institute, National Institutes of Health, Bethesda, MD USA; 7 Molecular Cytogenetics Core Facility, Mouse Cancer Genetics Program, Center for Cancer Research, National Cancer Institute, National Institutes of Health, Frederick, MD USA

**Keywords:** *Brca1*, *Trp53*, Rb, Breast cancer, Mouse model, Basal-like, Luminal B, Endocrine resistant

## Abstract

**Background:**

Breast cancer is a heterogenous disease with several histological and molecular subtypes. Models that represent these subtypes are essential for translational research aimed at improving clinical strategy for targeted therapeutics.

**Methods:**

Different combinations of genetic aberrations (*Brca1* and *Trp53* loss, and inhibition of proteins of the Rb family) were induced in the mammary gland by injection of adenovirus expressing Cre recombinase into the mammary ducts of adult genetically engineered mice. Mammary tumors with different genetic aberrations were classified into molecular subtypes based on expression of molecular markers and RNAseq analysis. In vitro potency assays and Western blots were used to examine their drug sensitivities.

**Results:**

Induction of *Brca1* and *Trp53* loss in mammary ductal epithelium resulted in development of basal-like hormone receptor (HR)-negative mammary tumors. Inhibition of Rb and *Trp53* loss or the combination of Rb*, Trp53* and *Brca1* aberrations resulted in development of luminal ductal carcinoma positive for ER, PR, and Her2 expression. HR positivity in tumors with Rb*, Trp53* and *Brca1* aberrations indicated that functionality of the Rb pathway rather than *Brca1* status affected HR status in these models. Mammary tumor gene expression profiles recapitulated human basal-like or luminal B breast cancer signatures, but HR-positive luminal cancer models were endocrine resistant and exhibited upregulation of PI3K signaling and sensitivity to this pathway inhibition. Furthermore, both tumor subtypes were resistant to CDK4/6 inhibition.

**Conclusions:**

Examination of molecular expression profiles and drug sensitivities of tumors indicate that these breast cancer models can be utilized as a translational platform for evaluation of targeted combinations to improve chemotherapeutic response in patients that no longer respond to hormone therapy or that are resistant to CDK4/6 inhibition.

**Supplementary Information:**

The online version contains supplementary material available at 10.1186/s13058-022-01566-4.

## Background

Breast cancer (BC) is the most commonly diagnosed cancer in women, and the second leading cause of cancer deaths [1]. BC is a heterogenous disease that can be histologically classified into several subtypes, with invasive ductal carcinoma being the most frequent, accounting for over 75% of all cases [2]. Recent improvements in the molecular classification of BC have allowed for the selection of more patient-tailored therapies [3]. Based on expression of molecular markers, all BC can be classified into several molecular subtypes: basal-like (ER^−^, PR^−^, Her2^−^, K5/14^+^, EGFR^+^), Her2 enriched (ER^−^, Her2^+^), claudin low (ER^−^, claudin^−^, vimentin^+^, E-cadherin ^low^, Zeb1^+^), luminal A (ER ^high^, Her2 ^low^), luminal B (ER ^low^, Her2 ^low^, proliferation ^high^) and normal breast-like (adipose tissue gene signature). Luminal A and B are the only hormone receptor (HR)-positive subtypes.

**Fig. 1:**
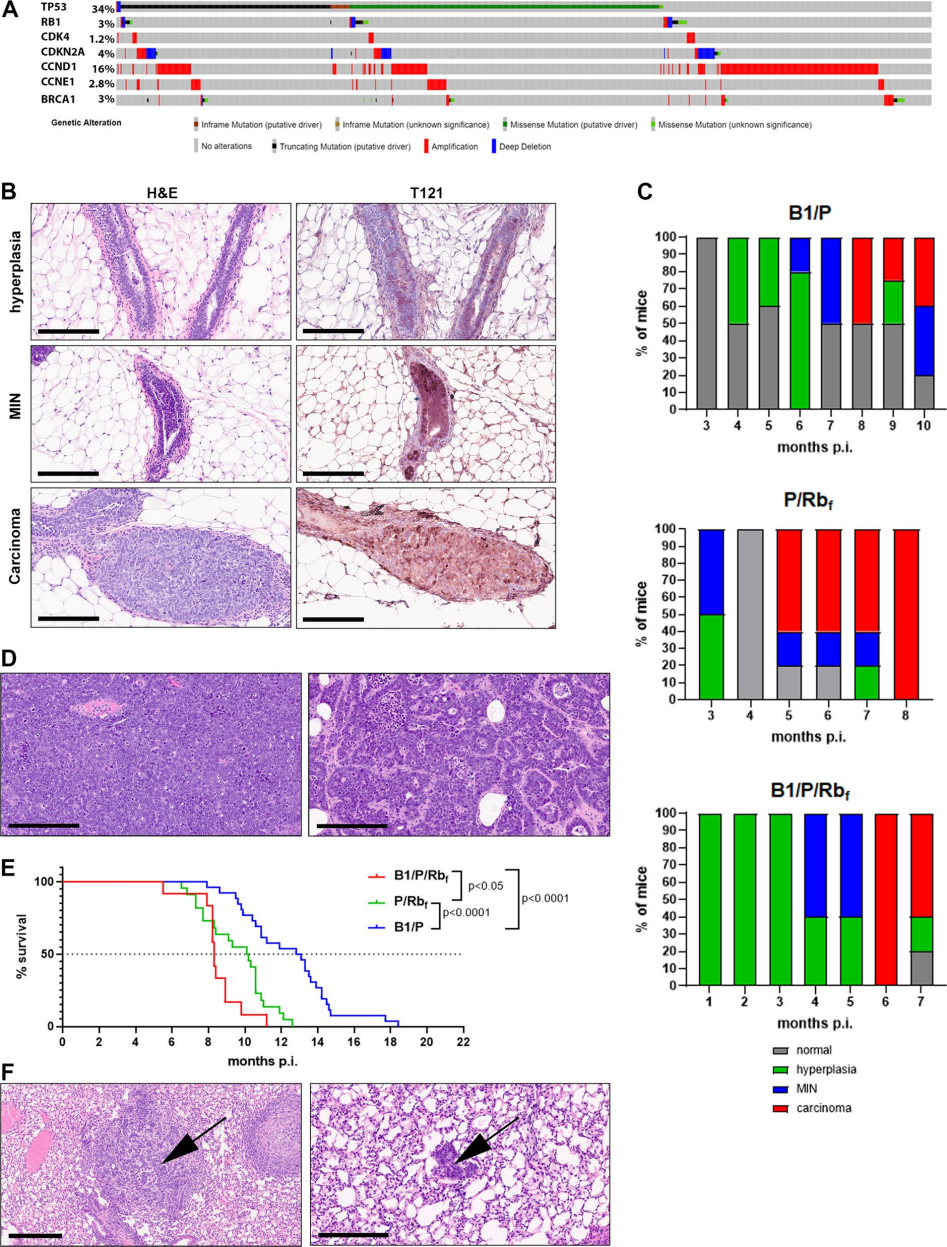
Development and characterization of mammary cancer models. **A** Oncoprint for somatic aberrations in *TP53*, *BRCA1* and genes of the Rb pathway in METABRIC cohort of primary breast cancer samples. Data were analyzed on cBioPortal web site and included all 2509 patients (Curtis, Shah et al. 2012, Pereira, Chin et al. 2016, Rueda, Sammut et al. 2019). **B** H&E and IHC for T121 on mammary ductal hyperplasia, MIN and carcinoma in B1/P/Rb_f_ mice. In early MINs, the epithelial cells lining ducts were hyperchromatic, with a small amount of cytoplasm, 2 or more layers of atypical cells and an increased mitotic rate. High-grade MINs had the additional criteria of greater cytologic and nuclear pleomorphism, and a further increased mitotic rate. Note focal expression of T121 (brown stain) in hyperplastic ductal epithelium. **C** Graphic representation of the spectrum of histological findings in induced mammary glands evaluated at various time points post-viral induction in all three genotypes (*N* = 5 mice per time point). **D** Example of solid and ductal histology of mammary adenocarcinoma in B1/P/Rb_f_ mice, **E** Kaplan–Meier survival plot for mice of three different genotypes. Statistical analysis performed by Log-rank (Mantel-Cox) test, **F** Adenocarcinoma histology of the occasional lung metastases (arrows). Scale bar 200 µm

BC is heterogeneous in its mutational profile, although certain genes and pathways are frequently affected [4–7]. Mutations in the Tumor Protein 53 (*TP53*) gene are observed in about 37% of patients [4], and while mutations in the retinoblastoma (*RB1*) gene occur in only 2.5% of invasive BC patients (Fig. 1A), the dysregulation of *RB1* and genes in the RB1 pathway, including Cyclin D1, p16Ink4a, CDK4, and Cyclin E, is observed in approximately 27% of patients (Fig. 1A). Just 3% of BC patients have genetic alterations in the Breast Cancer Type 1 susceptibility (*BRCA1*) gene (Fig. 1A), yet *BRCA1* mutation carriers have a 70% lifetime risk of developing breast cancer [8]. Moreover, *BRCA1* is an important marker to consider when selecting therapeutic regimens, as patients with *BRCA1* mutations have been shown to benefit from targeted therapy with PARP inhibitors [9]. Mouse models that incorporate aberrations in *Trp53, Brca1*, and the Rb pathway are thus important tools for preclinical research into therapeutics that may benefit patients.

Genetically engineered mouse models (GEMMs) for BC developed previously utilize transgenes for Cre recombinase driven by mammary-specific promoters, to drive loss of *Trp53* alone or in combination with loss of *Brca1* or *Rb1* in mammary tissue [10–13]. The most frequently used mammary-specific promoters for Cre transgene are keratin 14 (K14), whey acidic protein (WAP), bovine *β*-lactoglobulin (BLG) and mouse mammary tumor virus (MMTV), directing Cre expression into basal or myoepithelial cells, or luminal cells, that are mostly steroid-receptor negative [14–18]. Although these mammary-specific Cre transgenes are widely used, they do not allow for temporal control of the Cre expression and often result in induction of genetic events before onset of adulthood. Moreover, many of the Cre transgenes are expressed in other tissues in addition to the mammary gland, e.g., skin in K14-Cre mice [17], brain, testes and muscle in WAP-Cre mice [16], and multiple tissues in MMTV-Cre mice [18], thus complicating use of the models and data analysis.

The majority of tumors in promoter-driven transgenic models lack ER (estrogen receptor), PR (progesterone receptor) and Her2 (human epidermal growth factor receptor 2) expression. Up to 40% of WAP-Cre mice with loss of *Trp53* develop ER + tumors [11]; however, the extent of ER expression in these tumors is affected by specificity of the promoter driving the Cre transgene, targeting expression to different subpopulation of cells as well as into different percentage of cells, as indicated by differences between WAP-Cre and MMTV-Cre mice [11]. A more relevant model would initiate mammary cancer development in a promoter-independent, temporally controlled manner.

Here, we present new mouse models that develop mammary cancer with genetic aberrations frequently occurring in BC patients. To avoid a bias toward induction of genetic events in a specific mammary cell type and to control the timing of tumor initiation, we induced *Brca1* loss, inhibition of proteins of the Rb family (Rb_f_), and *Trp53* loss in the mammary ductal epithelium by injecting adenovirus expressing Cre recombinase into the mammary ducts of adult mice. We investigate three different allele combinations that cooperate to produce HR-positive or HR-negative mammary tumors of the luminal or basal-like subtype. Mice with *Brca1* and *Trp53* loss develop basal-like HR-negative mammary tumors, and mice with inhibition of proteins of the Rb family and *Trp53* loss or the combination of Rb, *Trp53* and *Brca1* aberrations develop luminal ductal carcinoma that is positive for ER, PR and Her2 expression, but endocrine resistant. Molecular characterization and drug sensitivities of the models point to pathways that may be exploited for comparisons of therapeutic response in these BC subtypes.

## Methods

### Experimental animals

NCI-Frederick is accredited by AAALAC International and follows the Public Health Service Policy for the Care and Use of Laboratory Animals. Animal care was provided in accordance with the procedures outlined in the “Guide for Care and Use of Laboratory Animals (National Research Council; 1996; National Academy Press; Washington, D.C.).” All study protocols were approved by the NCI at Frederick Animal Care and Use committee (Frederick, MD). Brca1^fl/fl^ (FVB;129- Brca1^tm1Brn^), p53^fl/fl^ (FVB;129- Trp53^tm1Brn^) mice were obtained from the NCI Mouse Repository (National Cancer Institute, Rockville, MD), and bred into C57Bl/6 J background. TgK18GT_121_^tg/+^ BAC transgenic mice were generated in-house [19] and were inbred on C57Bl/6 J background. C57Bl/6 J females were purchased from the Jackson Laboratory.

### Adenoviral induction

Recombinant adenovirus Ad5-CMV-Cre (Adeno-Cre) was purchased from the Viral Vector Core of the University of Iowa at a titer of 4 × 10^10^ pfu/ml. The #4 right side abdominal mammary glands of females were injected with 10 µl of undiluted virus via nipple. Animals were injected between 9 and 13 weeks of age. Tumor size was measured by caliper with end point set at 2 cm.

### Cell injections to establish allograft models

Single-cell suspensions of dissociated tumor cells from GEM models were injected into mammary glands of adult recipient C57Bl/6 female mice either through the nipple or directly into the mammary fat pad. Cells were resuspended in DMEM-F12 media at 50,000 or 100,000 cells per 10 µl.

### Hormone dependency

The allograft model was used to evaluate dependency of the tumor growth on estrogen and progesterone. Dissociated tumor cells were implanted into syngeneic C57Bl/6 recipient female mice via intraductal injection. To observe the growth of tumors in hormone free environment from the start, recipient mice were ovariectomized two weeks prior to tumor cell implantation. A group of implanted mice was also treated with tamoxifen once small tumors were established. Tamoxifen pellets (tamoxifen free base 0.5 mg pellet with 60-day slow release from Innovative Research of America, Sarasota, FL, USA) were implanted subcutaneously.

### Derivation of primary cell lines

To derive mammary carcinoma cell lines, primary tumors were dissociated into single cell suspension by incubating with 2 mg/ml collagenase IV (Worthington Biochemical Corporation, Lakewood, NJ, USA) in DMEM-F12 (Sigma Aldrich, St. Luis, MO, USA) under constant agitation at 37 °C for 1 h. Cells were strained through 100 µm cell strainer and washed with DMEM-F12. Cell pellets were resuspended in ACK lysis buffer (Thermo Fisher Scientific, Frederick, MD, USA) for 5 min to lyse red blood cells and then washed twice with PBS (Thermo Fisher Scientific, Frederick, MD, USA). Cells for in vitro assays were maintained in DMEM-F12 media supplemented with 100U/ml of penicillin and streptomycin and 0.25 µg/ml amphotericin B (Thermo Fisher Scientific, Frederick, MD, USA), 1 × insulin-transferrin-selenium (Thermo Fisher Scientific, Frederick, MD, USA), 2% fetal bovine serum (Thermo Fisher Scientific, Frederick, MD, USA), 0.4% bovine pituitary extract (Cell Applications, San Diego, CA, USA), 0.5 µg/ml hydrocortisone (Sigma Aldrich, St. Luis, MO, USA), 1 µg/ml hIGF1 (Prospec, Ness-Ziona, Israel) and 3 ng/ml EGF (Sigma Aldrich, St. Luis, MO, USA).

### Histology and immunohistochemistry (IHC)

Animals were euthanized by CO_2_ inhalation followed by cervical dislocation. Tissues were collected into 10% neutral buffered formalin for 48 h and processed for routine paraffin embedding. Five-micrometer sections were cut for hematoxylin and eosin staining and for immunohistochemical (IHC) stains. H&E-stained sections were evaluated by a board-certified veterinary pathologist (B.K., N.P. or L.B.). The specific conditions and antibodies used for IHC are detailed in Additional file 1: Table 1. 3,3’-Diaminobenzidine or Nova red was used to visualize peroxidase activity in IHC followed by hematoxylin counterstain. Stained slides were scanned using the Aperio AT2 digital whole brightfield slide scanner (Aperio, Vista, CA, USA) at 20 × magnification. IHC for ER, PR and Her2 were quantified by H-score, which accounts for number of stained cells as well as for the staining intensity as follows: H-Score = 3 × percentage of strongly staining nuclei + 2 × percentage of moderately staining nuclei + percentage of weakly staining nuclei, giving a range of 0 to 300, where 0–50 = negative result, 51–100 = mildly positive, 101–200 = moderate positive, > 200 = strong positive. All quantification was performed by digital image analysis of whole slide images using thoroughly validated algorithms in Aperio software. Regions of interest (ROIs) were annotated manually by a pathologist to exclude areas of necrosis and allow for automated quantification to identify the number of cells at each threshold of positivity. Results were reported as the absolute counts of stained cells with each intensity of expression including negative, low, moderate and high intensity cells. Rigorous quality control evaluation was performed by a pathologist. For Ki-67, validated Aperio algorithm was adapted by a veterinary pathologist to determine the total number of cells in the ROI as well as the number of cells with Ki-67 nuclear positivity. The percent Ki-67 positivity was determined by dividing the number of Ki-67 positive cells by the total number of cells.

### In vitro potency assays

Compounds for in vitro assays were obtained from Developmental Therapeutics Program at NCI (palbociclib, gedatolisib, afatinib, neratinib, buparlisib, alpelisib,), BEZ235 and trametinib from ChemieTek (Indianapolis, IN, USA), rapamycin from LC Laboratories (Woburn, MA, USA) and were dissolved in 100% DMSO. Primary mammary cancer cells were plated at 2,500 cells per well in opaque white 96-well plates. Single compound treatment was conducted at a range of concentrations between 0.1 and 50,000 nM in 0.5% DMSO. Compounds were added once to wells, 24 h after plating cells, and drug-treated wells were run in triplicate. At 72 h after drug exposure, cell viability was measured using CellTiter-Glo Luminescent Cell Viability Assay (Promega, Madison, WI, USA) as per the manufacturer’s instruction. DMSO-treated wells were considered as 100% viability for each treatment plate.

### RNA preparation, sequencing and analysis

Mammary glands and tumors were homogenized in TRIzol (Thermo Fisher Scientific, Frederick, MD, USA) using TissueLyser (Qiagen, Germantown, MD, USA). Chloroform was added to the cleared lysates and after separation of phases the clear aqueous phase was mixed with isopropanol to precipitate the RNA. RNA in isopropanol was loaded onto RNeasy columns (Qiagen, Germantown, MD, USA) and purified per manufacturer’s protocol. Total RNA was submitted for sequencing. Eighteen mRNA-Seq samples were pooled and sequenced on NovaSeq 6000 SP using Illumina Stranded mRNA Prep and paired-end sequencing. The sequencing quality of the reads was assessed per sample using FastQC (version 0.11.5)[20], Preseq (version 2.0.3)[21], Picard tools (version 1.119) (https://broadinstitute.github.io/picard/) and RSeQC (version 2.6.4)[22]. Sequencing reads were trimmed of low-quality bases, and adapter sequences were removed using Cutadapt (version 1.18)[23]. The trimmed reads were aligned using the GRCh38 (GENCODE hg38, version 30). Gene expression levels were quantified using RSEM (version 1.3.0) DEseq2 (version 1.28.1)[24]. Raw read counts (expected counts from RSEM) were imported into the NIH Integrated Data Analysis Platform for downstream analysis (https://nidap.nih.gov/). Counts were normalized to library size as log_2_CPM. Genes with a log_2_ count-per-million (CPM) ≥ 6 in at least 6 samples were analyzed. Samples were further normalized using the voom algorithms [25] quantile normalization from the Limma R package (v3.40.6) [26]. Differentially expressed gene (DEG) analysis was performed using Limma and pre-ranked gene set enrichment analysis (GSEA)[27] was performed using the KEGG [28] and REACTOME [29] databases. Genes or gene sets with an FDR adj. *p* value of ≤ 0.05 were considered statistically significant. Ingenuity Pathway Analysis software (Qiagen) was used to evaluate pathway perturbations and diagram results.

### Statistics

Results were expressed as means ± SD. Statistical analyses were performed with Prism 9.0 (GraphPad Software) and consisted of one-way ANOVA, followed by Tukey’s multiple comparisons test. Survival curves were compared by Log-rank (Mantel-Cox) test.

## Results

### Induction of aberrations in ***Trp53, Brca1*** and Rb_f_ in mammary ductal epithelium leads to development of mammary adenocarcinoma

The Cre-dependent *TgK18GT*_*121*_ transgene was designed to express 121 amino acids of the N-terminal region of the SV40 large T antigen under the keratin 18 (K18) promoter, inhibiting all 3 pocket proteins of the Rb family (pRb, p107, and p130; henceforth referred to as Rb_f_), specifically in epithelial cells [19]. We crossed *TgK18GT*_*121*_ mice to mice carrying *Brca1*^*fl/fl*^ and *Trp53*^*fl/fl*^ conditional alleles. In order to generate mice with genetic aberrations specifically in the mammary gland, we injected adenovirus expressing Cre recombinase through the nipple into the mammary ducts of 9–13-week-old female mice, thereby abrogating the need for a tissue-specific promoter restricted to adult expression.

Genetic events induced by Cre recombinase occurred in epithelial cells of the mammary ducts, as confirmed by IHC stain for T121 (Fig. 1B), in mice carrying *TgK18GT*_*121*_ transgene. We collected mammary glands at various time points after adenoviral injection to evaluate the development of lesions (Fig. 1C). The first histological changes in the transformed epithelium were epithelial hyperplasia and mammary intraepithelial neoplasia (MIN). Hyperplasia presented as epithelial ducts and glands multifocally lined by multiple layers of epithelial cells (Fig. 1B). Inflammatory cells were often present surrounding the ducts. In MIN, the epithelial cells lining ducts were hyperplastic and were composed of small, dark, cuboidal to low columnar cells forming papillary and glandular proliferation (Fig. 1B). Cells displayed anisocytosis and anisokaryosis with high mitotic rate. We compared the timelines for development of lesions in *Brca1*^*fl/fl*^*/Trp53*^*fl/fl*^*/TgK18GT*_*121*_^*Tg/*+^ (B1/P/Rb_f_) mice with *Brca1*^*fl/fl*^*/Trp53*^*fl/fl*^ (B1/P) and *Trp53*^*fl/fl*^*/TgK18GT*_*121*_^*Tg/*+^ (P/Rb_f_) mice (Fig. 1C). B1/P/Rb_f_ mice displayed hyperplastic changes as early as 1-month post-induction (p.i.) while hyperplasia was not observed until 3–4 months p.i. in B1/P and P/Rb_f_ mice. MINs progressed to adenocarcinoma as early as 6 months p.i. in B1/P/Rb_f_ mice, and as late as 8 months p.i. in B1/P mice (Fig. 1C).

Adenocarcinomas developed multifocally in all three models and progressed to form partially circumscribed, non-encapsulated, multi-lobulated, nodular masses effacing and replacing the normal gland. Neoplastic cells were arranged in densely cellular sheets or formed poorly defined tubular structures (Fig. 1D). The majority of tumors were characterized as ductal or solid adenocarcinomas (Fig. 1D). A subset of tumors in B1/P mice exhibited heterogeneity in the adenocarcinoma tumor histology, developing metaplastic carcinomas with foci of squamous differentiation (adenosquamous carcinoma) in 14% of tumors (5/36), and occasionally tumors with mesenchymal differentiation (6%, 2/36). In comparison, all B1/P/Rb_f_ mice developed mammary adenocarcinoma, and only one mouse in the P/Rb_f_ cohort developed carcinoma with focal mesenchymal differentiation.

The survival of tumor-bearing mice (based on tumor growth end point) was dependent on the genotype, with the shortest survival observed in B1/P/Rb_f_ mice (*N* = 12, mean survival 8.5 months p.i.). The mean survival in P/Rb_f_ mice (*N* = 22) was 9.5 months p.i., and the longest survival was observed in B1/P mice (*N* = 26, mean survival 12.7 months p.i.) (Fig. 1E). Models occasionally developed lung metastases (Fig. 1F), however, no bone or brain metastases were observed. Recombination of floxed alleles was confirmed by PCR in all mammary tumors, and loss of Brca1 expression was confirmed in B1/P/Rb_f_ and B1/P tumors by RT-qPCR (Additional file 2:. Figure 1).

### Inhibition of Rb_f_, not *Brca1* status, determines molecular subtype of mammary adenocarcinoma

**Fig. 2:**
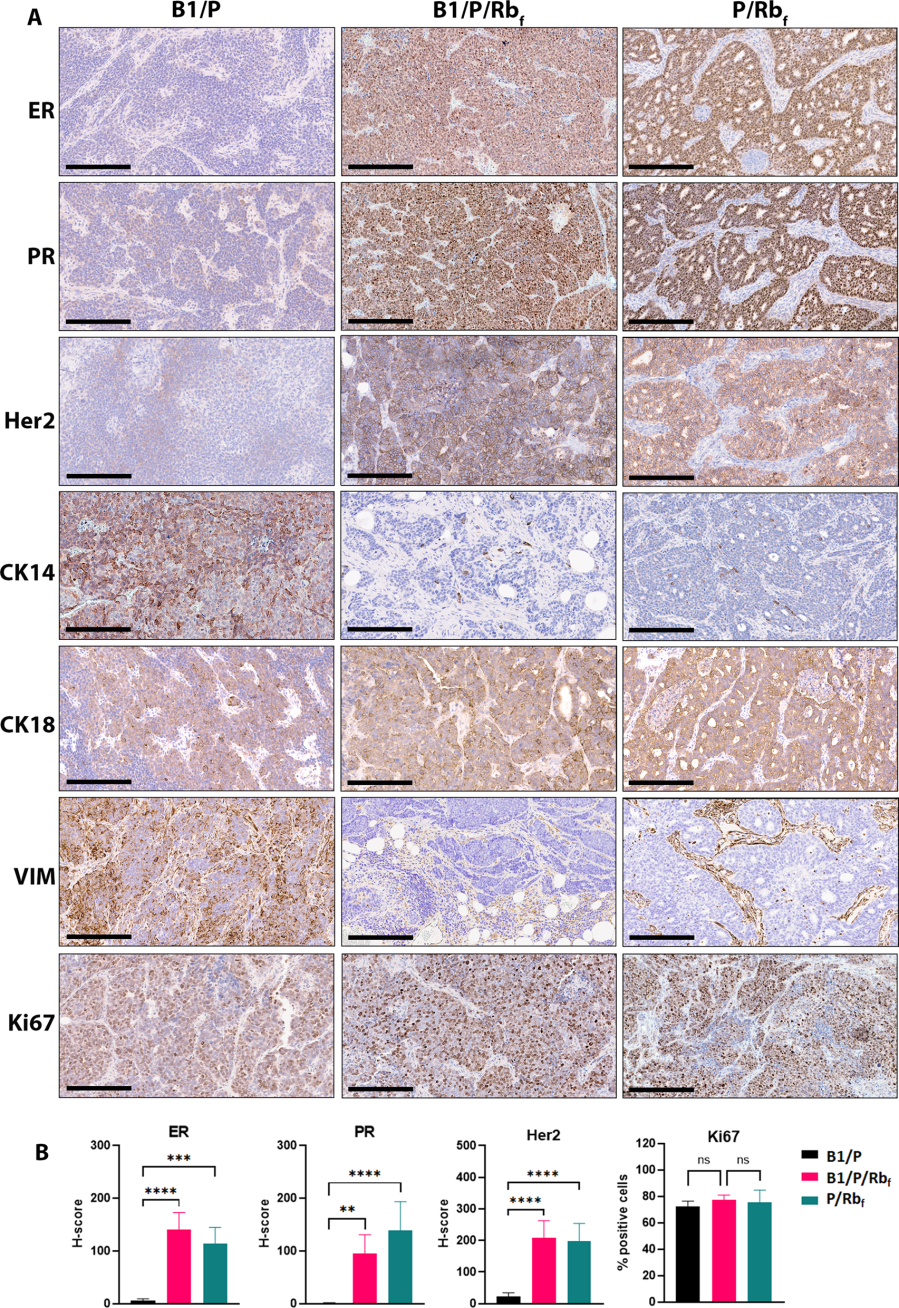
Analysis of molecular marker expressions in mammary tumors of three different genotypes. **A** Representative ER−, PR−, HER2−, CK14 + , CK18 + and VIM + staining in B1/P mammary tumors and ER + , PR + , HER2 + , CK18 + , CK14−_and VIM- staining in B1/P/Rb_f_ and P/Rb_f_ mammary tumors. B1/P/Rb_f_ tumors displayed the highest expression of Ki67. ER = estrogen receptor, PR = progesterone receptor, Her2 = human epidermal growth factor receptor 2, CK14 = cytokeratin 14, CK18 = cytokeratin 18, VIM = vimentin, Ki67 = marker of proliferation Ki-67. Expression is indicated by DAB positivity (brown stain). **B** Quantitative analysis of IHC stains in tumors. Mean and SD are plotted, one-way ANOVA was used for statistical analysis. B1/P/Rb_f_ tumors *N* = 8, P/Rb_f_ tumors *N* = 6, B1/P tumors *N* = 5. Scale bar 200 µm

Human BC is classified into subtypes based on molecular and histological properties, and hormone receptor status is a major determinant for therapeutic options. Tumors from B1/P mice were negative for ER and PR, as well as Her2 expression (Fig. 2A–B), similar to other triple-negative models with *Brca1* and *Trp53* loss that have been described previously [30]. Status of hormone receptor expression was also assessed by quantitative RT-PCR for *Esr1* and *Pgr* mRNA, which confirmed the lack of expression of both receptors (Additional file 3: Figure 2). B1/P tumors also expressed vimentin and cytokeratin 14 (assessed by IHC), indicating resemblance to the triple-negative, basal-like subtype of BC in humans (Fig. 2A). These tumors also exhibited widespread positivity for p63, and histology was consistent with adenocarcinoma (Additional file 4: Figure 3).

In contrast, inhibition of the Rb family proteins in B1/P/Rb_f_ and P/Rb_f_ mice resulted in development of triple positive mammary tumors with high expression of ER, PR and HER2 (Fig. 2A–B). Fluorescence in situ hybridization (FISH) in cell lines derived from B1/P/Rb_f_ primary tumors revealed an additional copy of *Erbb2* (*Her2*) on chromosome 14 in addition to the endogenous copy on chromosome 11 (Additional file 5: Figure 4). B1/P/Rb_f_ tumors were positive for the epithelial marker cytokeratin 18 (CK18), however, cytokeratin 14 was also expressed by a subpopulation of cells (0–30% of cells) indicating a variable component of cells with basal differentiation in tumors (Fig. 2A). Tumor cells were negative for vimentin while stromal cells surrounding tumors were highly positive for this marker (Fig. 2A). Based on the ER + , PR + , HER2 + , Vimentin −, and CK18 + molecular profile, B1/P/Rb_f_ tumors represent luminal type B breast cancers with a high proliferative index. P/Rb_f_ tumors with intact *Brca1* were also ER + , PR + , Her2 + , Vimentin -, and CK18 + (Fig. 2A–B). Therefore, although all 3 models were induced in the same manner in the ductal epithelium, B1/P mice develop HR- basal-like tumors while B1/P/Rb_f_ and P/Rb_f_ mice develop HR + luminal tumors. Tumors of all three genotypes were highly proliferative with B1/P/Rb_f_ tumors exhibiting an average of 77% cells positive for Ki67, triple-negative B1/P tumors 72% and P/Rb_f_ tumor 75% (Fig. 2A–B).

**Fig. 3:**
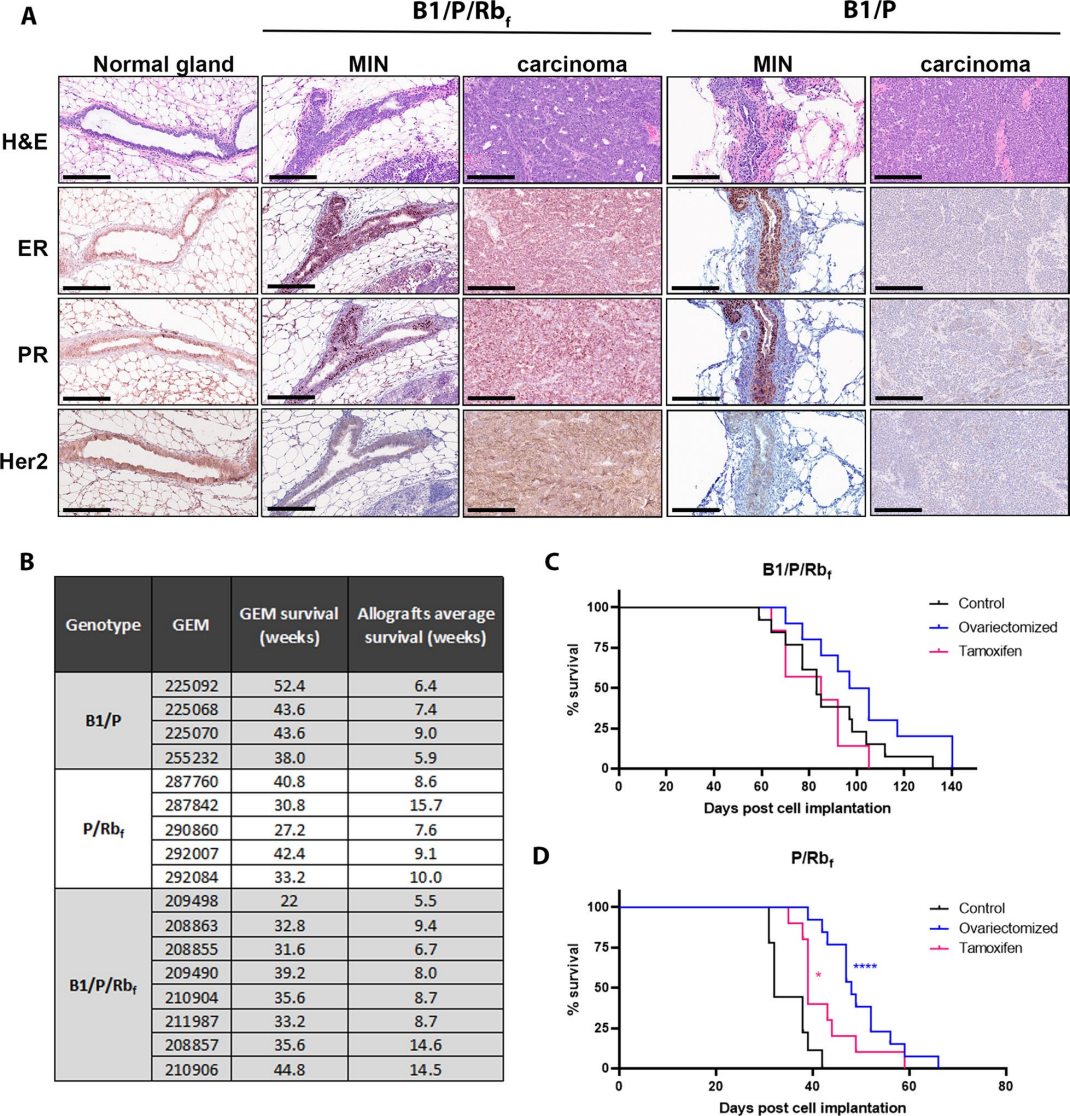
Hormone receptor status changes during GEM mammary tumor progression depending on genotype and is maintained in orthotopic tumors. **A** Loss of hormone receptor expression in B1/P model occurs after progression from MIN to carcinoma stage. Comparison of the hormone receptor expression in normal gland, in early and in late-stage lesions in B1/P/Rb_f_ and B1/P models is shown. Scale bar 200 µm. **B** Summary of allograft lines derived from GEM tumors and comparison of their latencies to GEM models. Latencies for allografts are averages from *N* = 5 mice. **C**–**D** Growth of ER + allograft tumors was not inhibited by tamoxifen treatment, or by implanting tumor cells into pre-ovariectomized mice. **C** B1/P/Rb_f_ cells (passage 1) from the 210,904 tumor line were implanted intraductally into strain-matched recipient mice and treated with tamoxifen, or were implanted into ovariectomized recipients. Differences in survival were not significant by the Log-rank (Mantel-Cox) test, **D** P/Rb_f_ cells (passage 2, freshly dissociated cells) from the 290,860 tumor line were implanted intraductally into strain-matched recipient mice and treated with tamoxifen, or were implanted into ovariectomized recipients. Ovariectomized mice survived longer than controls (the difference was significant by the Log-rank (Mantel-Cox) test), but all tumors grew to endpoint

The lack of HR expression in B1/P tumors prompted us to compare marker expression at early stages of cancer development in each model. We found that ER, PR and HER2 expression were retained in luminal cells during progression of the disease through hyperplasia and MIN in all genotypes; however, progression to carcinoma in B1/P mice resulted in loss of expression (Fig. 3A). These results suggest that HR-negative and HR-positive BC in these models could arise from a common luminal progenitor population, and loss or retention of HR expression upon progression to cancer is affected by status of the Rb pathway.

### Orthotopic allograft models recapitulate the original GEMM and are hormone-independent

To assess whether growth of ER + luminal carcinomas was dependent on presence of sex hormones, we first established an orthotopic allograft model by injecting freshly dissociated tumor cells from B1/P/Rb_f_ or P/Rb_f_ models directly into the mammary ducts of wildtype strain-matched female mice. Allografts recapitulated the histology as well as the expression of all the molecular markers of the primary GEMM tumors (Additional file 6: Figure 5A and B). Injected cells established tumors with considerably shortened latency compared to the GEM models (Fig. 3B). Survival (based on tumor growth end point) was tumor line dependent, averaging 5–15 weeks in allograft models compared to 20–52 weeks (5–13 months) in the GEMMs. When B1/P/Rb_f_ allograft tumor-bearing recipients were treated with tamoxifen, no growth inhibition or regression was observed compared to untreated mice (median survival 85 days vs. 83 days, respectively, *p* = 0.55: Fig. 3C). Mice that were ovariectomized 2 weeks prior to cell implantation supported tumor growth with only a slight delay compared to the control group (median survival 101 days vs. 83 days in control, *p* = 0.08: Fig. 3C). Survival of P/Rb_f_ allograft models was relatively increased with tamoxifen treatment: median survival was 39 days, compared to 32 days in control mice, *p* = 0.0048 (Fig. 3D). Median survival of ovariectomized P/Rb_f_ tumor-bearing mice was 48 days compared to 32 days in non-ovariectomized (*p* < 0.0001) (Fig. 3D). Despite the increase in survival of ovariectomized or tamoxifen-treated mice, all tumors grew to endpoint size. Therefore, the tumors are functionally hormone-insensitive due to their continuous growth in the absence of hormone supplementation [31]. We conclude that, in spite of the HR-positive status of B1/P/Rb_f_ and P/Rb_f_ tumors, growth of tumors was not estrogen-dependent and was correspondingly refractory to hormone therapy with tamoxifen.

To investigate whether hormone status was dependent on continued tumor passage via intraductal injection only, we established allograft models by injection of GEM tumor cells into the mammary fat pad. Comparison of allografts derived by intraductal injection versus fat pad injection of cells revealed that the fat pad-injected allografts were very similar in terms of latency, histology and expression of molecular markers to tumors generated by intraductal injection (Additional file 7: Fig. 6A-B).

### HR-positive and HR-negative mammary tumors recapitulate human luminal and basal-like breast cancer expression signatures

**Fig. 4:**
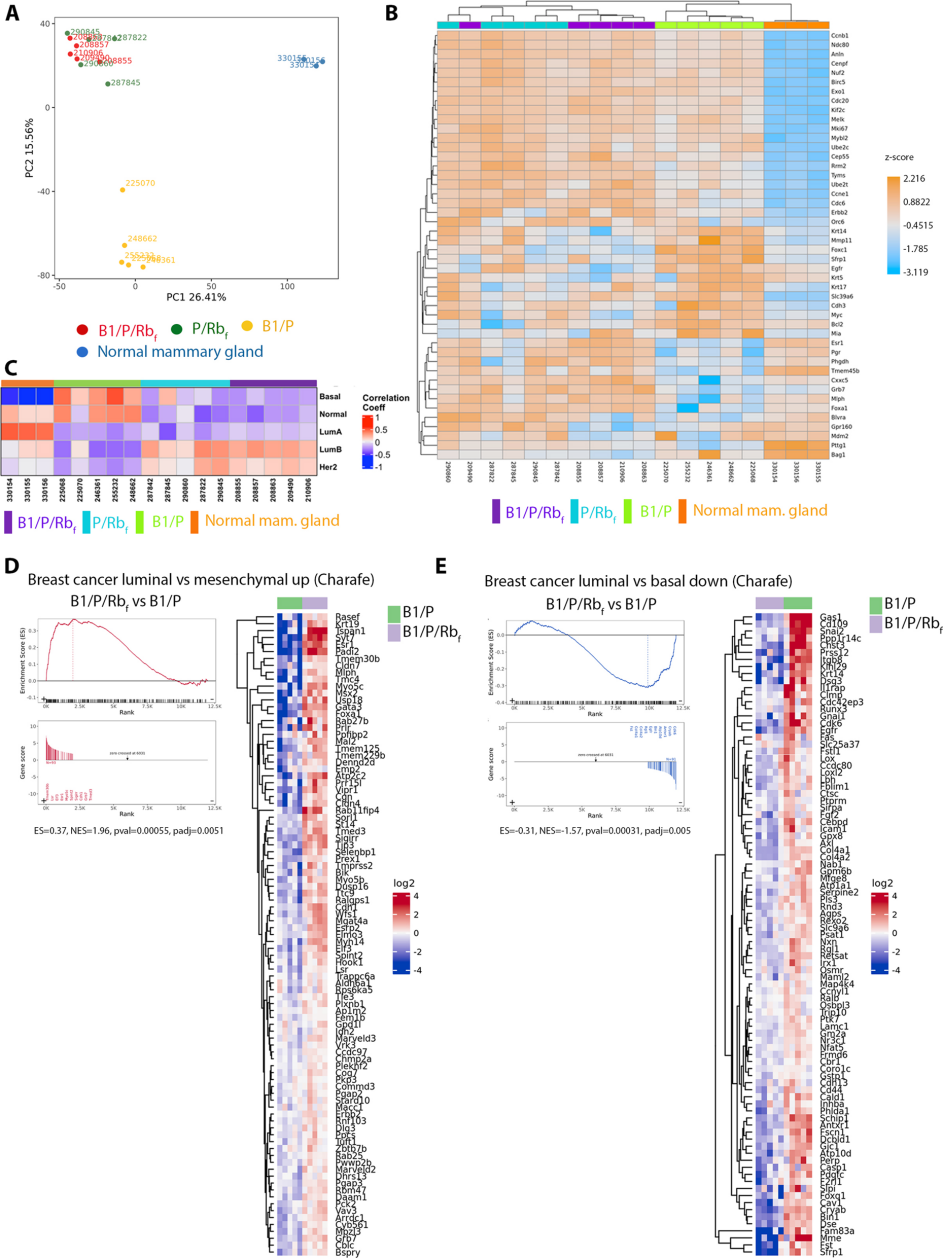
Gene expression analysis reveals segregation of mammary tumors into patient-defined luminal and basal-like molecular subtypes. **A** Principal component analysis shows that samples clustered into 3 separate groups, normal mammary glands (blue), B1/P tumors (yellow), and tumors with Rb_f_ inhibition (red, green). **B** Hierarchical clustering of PAM50 genes divides B1/P tumors from tumors with Rb_f_ inhibition. **C** Correlation analysis with PAM50 signatures indicates high association of B1/P tumors with the basal-like subtype and tumors with Rb_f_ inhibition with the luminal B subtype. **D**–**E** GSEA shows significant enrichment for curated gene sets distinguishing luminal vs basal-like breast cancer. **D** The gene set upregulated in luminal vs mesenchymal breast cancer is highly expressed in B1/P/Rb_f_ tumors compared to B1/P tumors. **E** The gene set downregulated in luminal vs basal breast cancer is suppressed in B1/P/Rb_f_ tumors compared to B1/P tumors

In addition to subtype differentiation by histology markers, the heterogeneity of BC has been described at the molecular level by gene expression classification [32]. To evaluate whether histology subtypes in the three mammary tumor models were reflected in their gene expression profiles, we analyzed RNA-seq data from a set of five tumors from each model and three normal strain-matched mammary glands. Principal component analysis (PCA) (Fig. 4A) as well as an unsupervised hierarchical clustering of genes (Additional file 8: Fig. 7A) segregated samples into three distinct groups: normal mammary gland, B1/P tumors, and a third group comprised of B1/P/Rb_f_ and P/Rb_f_ tumors, confirming that the histology differences evidenced by these genotypes were reflected in their gene expression profiles (Fig. 4A, Additional file 8: Figure 7A). Hierarchical clustering of tumors based on gene expression of a 50-gene subtype predictor developed using expression data from human breast cancer prototype samples (Prediction Analysis of Microarray 50, PAM50) [33], also separated B1/P/Rb_f_ and P/Rb_f_ tumors from B1/P tumors (Fig. 4B). A correlation coefficient between PAM50 subtype centroids and the gene expression for each mouse tumor sample was calculated (Fig. 4C). This analysis confirmed that B1/P/Rb_f_ and P/Rb_f_ tumors were most similar to human luminal B cancers, and B1/P tumors to the basal-like subtype.

**Fig. 5:**
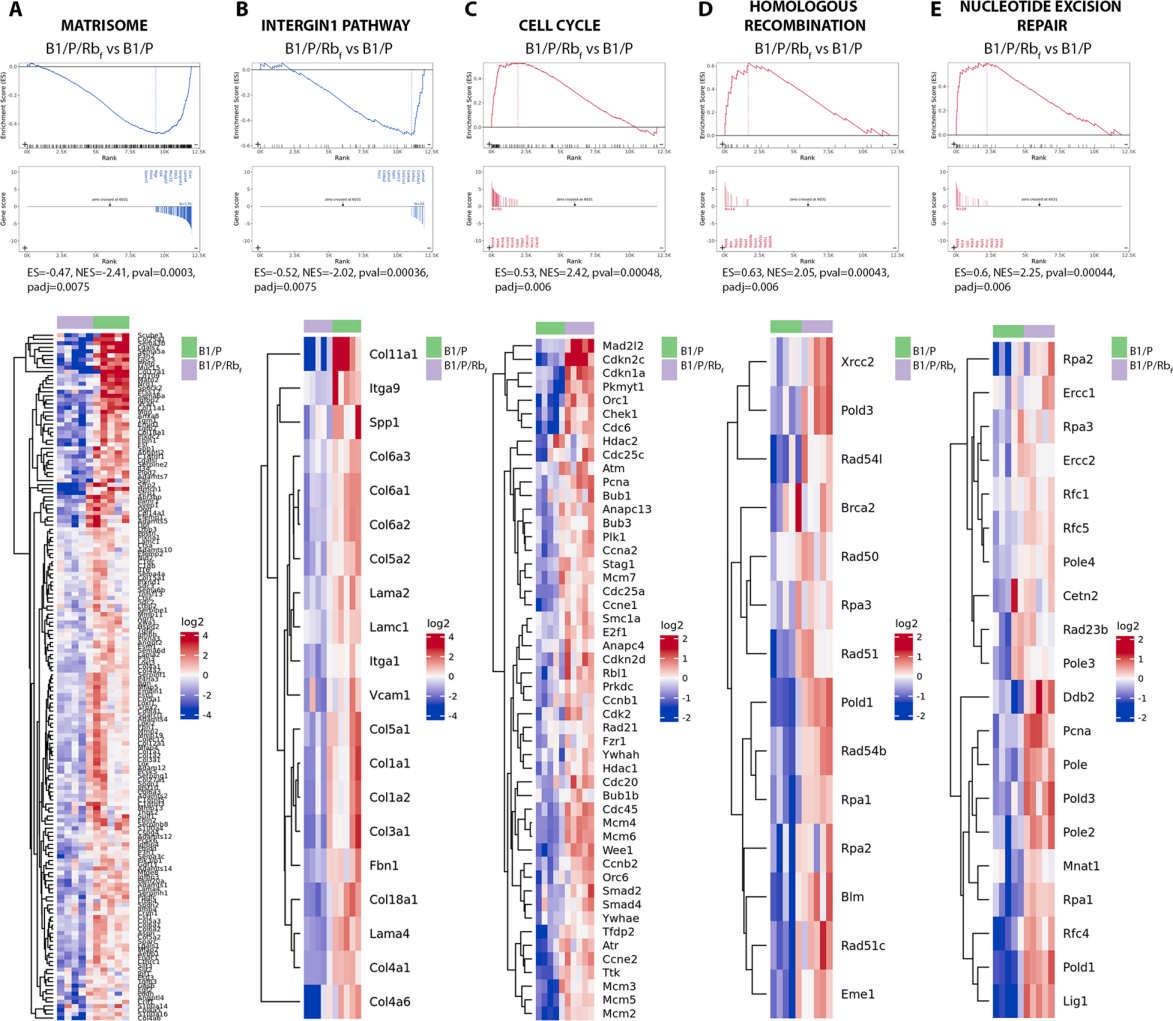
Several examples of pathways enriched in GSEA comparing B1/P/Rb_f_ and B1/P models. **A**-**B**) Genes of the matrisome **A** and integrin **B** gene set were upregulated in B1/P compared to B1/P/Rb_f_ tumors. **C**–**E**) Genes of the cell cycle **C**, homologous recombination **D** and nucleotide excision repair **E** were upregulated in B1/P/Rb_f_ compared to B1/P tumors

As the subtype distinctions have both prognostic and predictive clinical value, we applied gene set enrichment analysis (GSEA) to the differentially expressed genes (DEGs) in B1/P/Rb_f_ compared to B1/P tumors (Additional file 9: Tables 2 and Additional file 10: Table 3). Curated gene sets that were reported to distinguish luminal from basal or mesenchymal BC in human cell lines [34, 35] were significantly enriched in the mammary tumors (Additional file 10: Table 3, Fig. 4 D–E). B1/P/Rb_f_ tumors had increased expression of genes that were previously identified as upregulated in the luminal subtype of BC [35], including *Esr1* (estrogen receptor), *Krt19* (cytokeratin 19), zinc finger transcription factor *Gata3* and Forkhead box protein A1, *Foxa1* (Fig. 4D). Conversely, a gene set with reduced expression in the luminal vs. mesenchymal subtype in human cell lines [35] was likewise reduced in B1/P/Rb_f_ tumors compared to B1/P tumors (Fig. 4E). Further analysis revealed that B1/P basal-like tumors were enriched for matrisome and integrin gene sets [36, 37] with increased expression of collagens, laminins, integrins and extracellular matrix remodeling proteases (Fig. 5A–B). They also had decreased expression of genes involved in cell cycle regulation compared to B1/P/Rb_f_ tumors (Fig. 5C and Additional file 8: Fig. 7B), including mitotic checkpoint genes *Cdc20*, *Bub1*, and *Bub3*, corresponding to the slower growth of tumors observed in vivo (Fig. 1E). Although *Brca1* was depleted in both basal-like B1/P and luminal B1/P/Rb_f_ models (Suppl. Figure 1D), expression of homologous recombination and nucleotide excision repair genes was comparatively lower in B1/P tumors (Fig. 5D-E), as expected for the triple-negative subtype of breast cancer.

**Fig. 6:**
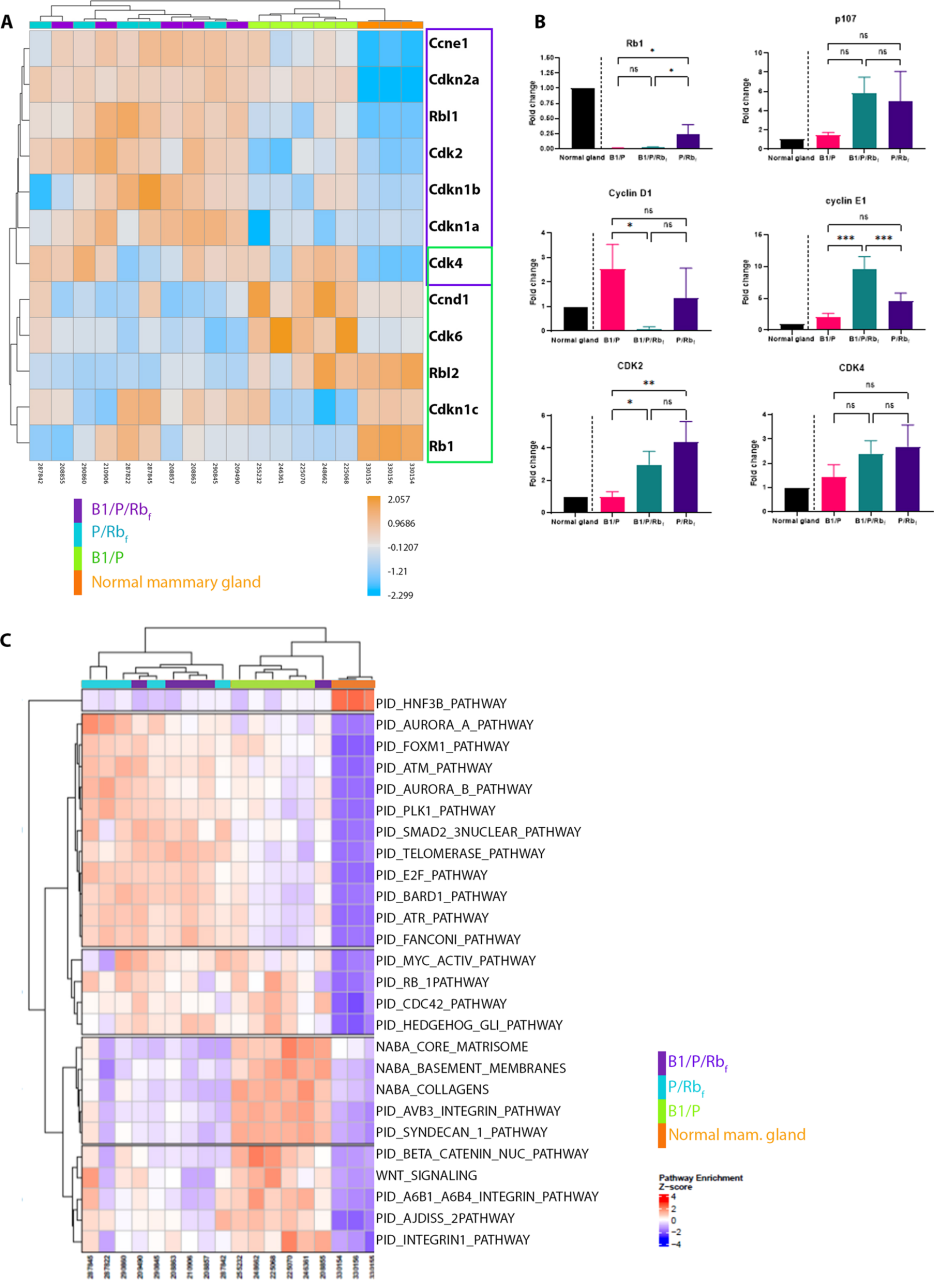
Pathway analysis reveals key differences in Rb pathway components and upregulation of druggable targets in mammary tumors. **A** Clustering of normalized RNA-seq data for genes in the Rb pathway revealed low levels of *Rb1* mRNA in the majority of tumors (regardless of genotype), and additional dysregulation of the pathway by increased expression of *Ccne1* (Cyclin E), *Cdkn2A (*Cyclin-dependent kinase inhibitor 2A; p16)*, Rbl1* (Retinoblastoma-like 1; p107)*, Cdk2* (Cyclin-dependent kinase 2), *Cdkn1b* (Cyclin-dependent kinase inhibitor 1B, p27)*, Cdkn1a* (Cyclin-dependent kinase inhibitor 1A; p21) and *Cdk4* (Cyclin-dependent kinase 4) (highlighted in purple frame) in tumors with Rb_f_ inhibition compared to the normal mammary glands and increased expression of *Ccnd1* (Cyclin D1)*, Cdk4, Rbl2* (Retinoblastoma-like 2; p130) and *Cdk6* (Cyclin-dependent kinase 6) (highlighted green frame) in B1/P tumors. **B** Expression of genes in the Rb pathway was analyzed by RT-qPCR on a set of 3 to 5 tumors from each genotype. Expression was normalized to normal mammary gland shown on the left side of each graph for illustration. Comparison of expression was performed between tumors of different genotype using One-tail ANOVA statistical analysis. **C** Single-sample GSEA (ssGSEA) heatmap highlights canonical pathways that were significantly enriched in mammary tumors

Hormone receptor positivity in mammary tumors was not correlated to the presence or absence of the *Brca1* allele, but instead to inhibition of Rb_f_ via expression of the *TgK18GT*_*121*_ allele, pointing to differences in Rb pathway regulation as a key determinant between HR-positive and HR-negative models. *Rb1*(pRb) gene expression was depleted in B1/P HR-negative tumors relative to the normal mammary gland (Fig. 6A and B), similar to observations in human triple-negative *BRCA1*-mutated tumors [38–40]. *Rb1* gene expression was also decreased in B1/P/Rb_f_ and P/ Rb_f_ HR-positive tumors (Fig. 6A-B), even though Rb family proteins were already suppressed at the protein level by T121. However, expression of Rb_f_ components p107 and p130, as well as expression of genes within the Rb pathway, differed between HR-positive and HR-negative tumors (Fig. 6A-B). Increased expression of cell cycle genes *Ccne1* (Cyclin E), *Cdkn2A* (Cyclin-dependent kinase inhibitor 2A; p16)*, Rbl1* (Retinoblastoma-like 1; p107)*, Cdk2* (Cyclin-dependent kinase 2), *Cdkn1b* (Cyclin-dependent kinase inhibitor 1B, p27)*, Cdkn1a* (Cyclin-dependent kinase inhibitor 1A; p21) and *Cdk4* (Cyclin-dependent kinase 4) was observed in Rb_f_ suppressed tumors compared to the normal mammary glands, and increased expression of *Ccnd1* (Cyclin D1)*, Cdk4, Rbl2* (Retinoblastoma-like 2; p130) and *Cdk6* (Cyclin-dependent kinase 6) characterized B1/P tumors (Fig. 6A, Additional file 11: Fig. 8A–B). Thus, while *Rb1* gene expression is decreased in tumors from both B1/P/Rb_f_ and B1/P models, we conclude that suppression of the protein family in B1/P/Rb_f_ results in dysregulation of the pathway through different mechanisms. Of note, resistance to CDK4/6 inhibition has been previously linked to activation of CDK2 and modification of CCNE1, MYC, and CDKN1A activity [41], genes that were overexpressed in the B1/P/Rb_f_ tumors.

### Expression of *MCM* and other genes involved in cell cycle regulation and progression differentiate B1/P/Rb_f_, P/Rb_f_ and B1/P tumors

Although B1/P/Rb_f_ and P/Rb_f_ tumors exhibited similar gene expression profiles when compared to normal mammary glands, GSEA on DEGs comparing the two HR-positive luminal models revealed that B1/P/Rb_f_ tumors had increased expression of genes of cell cycle, ribosome, nucleotide excision repair, and DNA replication gene sets compared to P/Rb_f_ tumors (Additional file 12: Table 4 and Additional file 13: Figure 9A–C). Notably, increased expression of genes in the minichromosome maintenance (Mcm) protein complex were common to the cell cycle and DNA replication gene sets that were differentially expressed in the two models (Additional file 12: Table 4; Additional file 13: Fig. 9D–E). The MCM complex, made up of six proteins, MCM2 through MCM7, is a DNA helicase essential for genomic DNA replication [42], and a direct association between MCM7 and proteins of the Rb family in vivo was shown previously to lead to inhibition of DNA replication [43]. In tumors with Rb_f_ suppression, release of MCM complex helicase activity thus may lead to increased DNA replication, as evidenced by decreased tumor latency observed in B1/P/Rb_f_ and P/Rb_f_ mice compared to B1/P mice. Several members of the Mcm family have been previously reported as highly expressed in malignancies including BC [44, 45], and have been proposed as prognostic markers and potential therapeutic targets [46, 47].

### Sensitivity of mammary tumors to targeted drugs

**Fig. 7:**
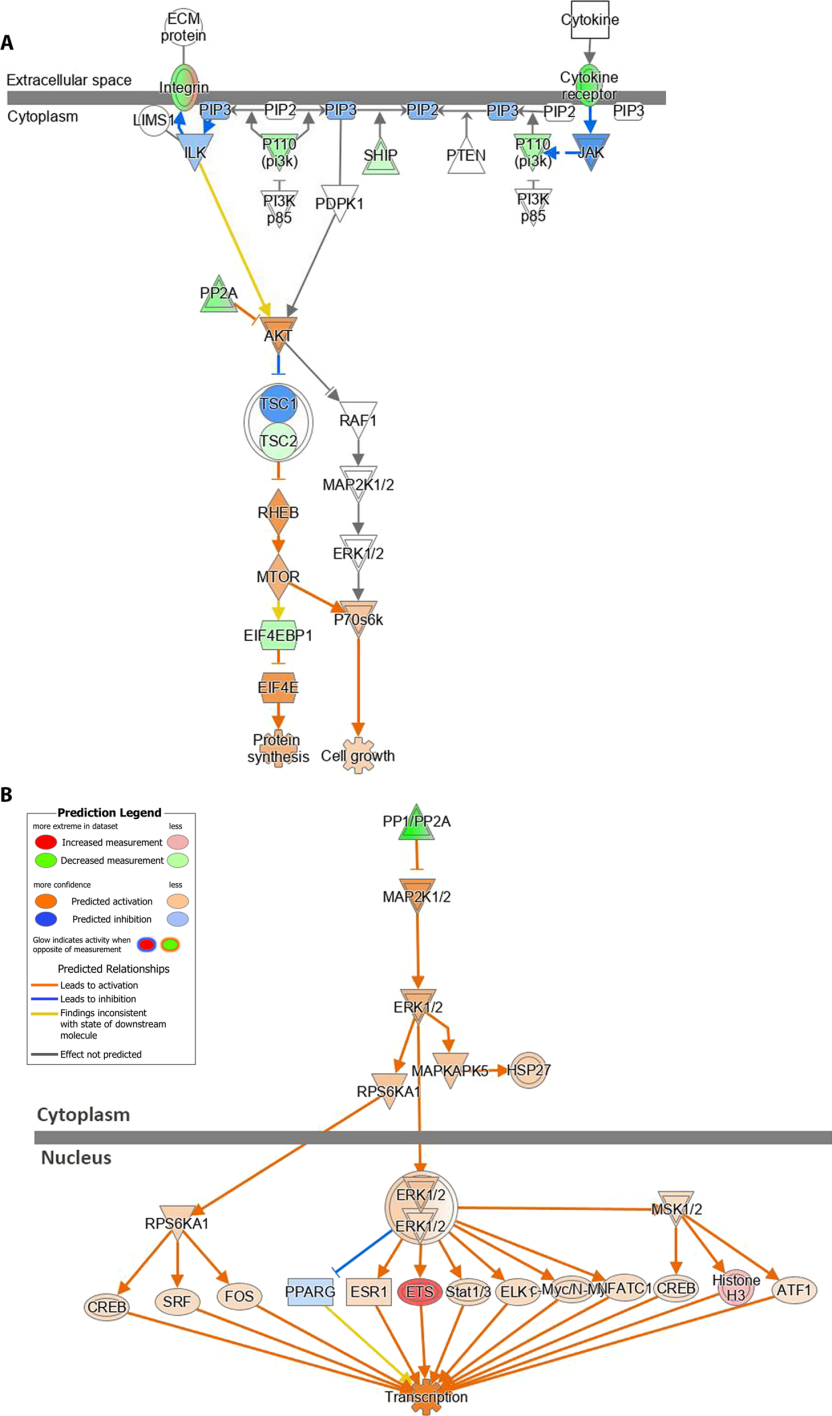
Upregulation of additional targetable pathways. Ingenuity Pathway Analysis diagrams illustrating alterations in PI3K/AKT **A** and ERK/MAPK **B** canonical pathways in B1/P/Rb_f_ tumors. Data were analyzed through the use of QIAGEN Ingenuity Pathway Analysis (*Bioinformatics*. 2014 Feb 15;30(4):523–30)

Luminal B1/P/Rb_f_ tumors were distinct from basal-like B1/P tumors by gene expression and HR-positivity status, yet they lacked sensitivity to hormone removal, and anti-hormonal therapy with tamoxifen was not effective. Thus, we explored alternate pathways for targeted therapeutics. GSEA analysis revealed several significantly enriched canonical pathways in mammary tumors (Additional file 14: Table 5). We used Single-sample GSEA (ssGSEA) to calculate separate enrichment scores for each pairing of a sample and a gene set, which were then plotted in a heatmap displaying pathway enrichment for each individual sample (Fig. 6C). ssGSEA in HR-positive tumors revealed enrichment for canonical pathways encompassing druggable targets, such as DNA repair, telomerase, aurora kinase, and PLK1 (Fig. 6C, Additional file 14: Table 5). Key components of the PI3K and MAPK pathways were also upregulated in all three tumor types (Fig. 7A–B).

**Fig. 8:**
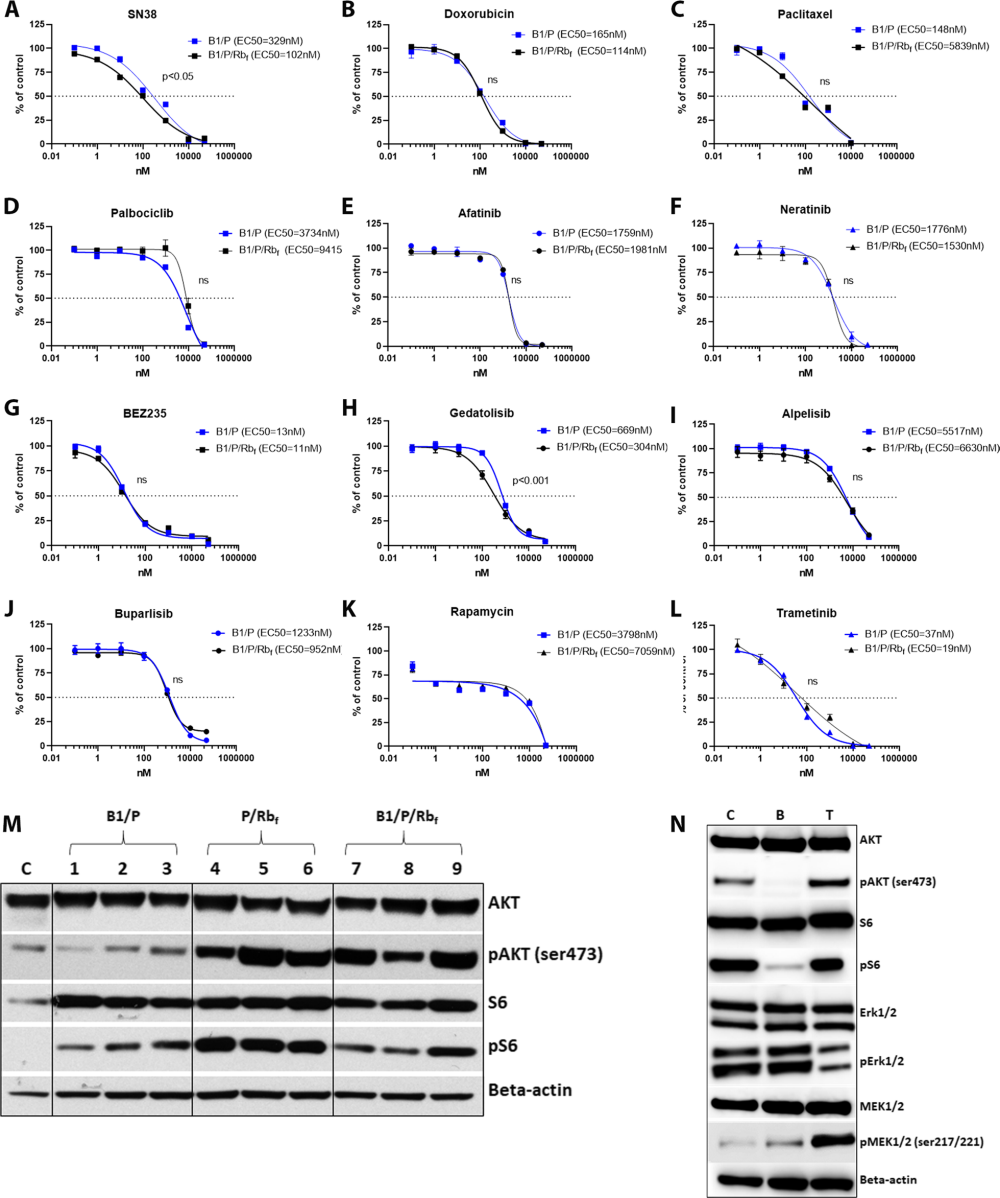
Mammary tumor cells respond to select targeted treatments. Black curves indicate potency in the B1/P/Rb_f_ cell line and blue curves potency in the B1/P cell line. Cells display sensitivity to chemotherapeutic agents, PI3K/mTOR dual inhibition, and MEK inhibition, and are resistant to CDK4/6 and EGFR/Her2 inhibition. **A** Topoisomerase I inhibitor, irinotecan/SN38. **B** Topoisomerase II inhibitor, doxorubicin. **C** Microtubule inhibitor, paclitaxel. **D** CDK4/6 inhibitor, palbociclib. **E**–**F**) EGFR/Her2 inhibitors, afatinib and neratinib. **G-H** PI3K/mTOR inhibitors, BEZ235 and gedatolisib. **I**–**J**) PI3K inhibitors, alpelisib and buparlisib. **K** mTOR inhibitor, rapamycin. **L** MEK inhibitor, trametinib. Differences between EC50 values were evaluated by extra sum-of-squares F test using Graph Pad Prism. **M** Western blot analysis shows activation of PI3K pathway by increased phosphorylation of AKT and S6 in mammary tumors of P/Rb_f_ and B1/P/Rb_f_ tumors compared to normal mammary gland, **N** Inhibition of PI3K and MEK pathways after 4 h of treatment with BEZ235 or Trametinib in B1/P/Rb_f_ cell line. *C* = control (DMSO-treated cells), *B* = BEZ235 (200 nM) treated cells, *T* = trametinib (200 nM) treated cells

To assess the efficacy of therapeutics targeting these pathways, mammary cell lines derived from the primary tumors from basal-like and luminal models were subjected to in vitro potency assays (Fig. 8A–L). Standard-of-care chemotherapeutic agents targeting cell division and DNA repair, including anthracyclines (doxorubicin; a topoisomerase II inhibitor), taxanes (paclitaxel; a microtubule inhibitor) and SN38 (the active metabolite of irinotecan; a topoisomerase I inhibitor), were potent in tumor cell lines derived from both models (Fig. 8A–C). The RB pathway was enriched in B1/P/Rb_f_ tumors (Fig. 6C), but cells were not sensitive to the CDK4/6 inhibitor, palbociclib (Fig. 8D), confirming that inhibition of Rb_f_ by T121 results in activation of the pathway downstream of CDK4/6/cyclin D1. B1/P cells were resistant to palbociclib as well, likely as a consequence of *Rb1* downregulation (Fig. 6A). The EGFR/Her2 inhibitors afatinib and neratinib were not potent in B1/P/Rb_f_ tumor cells despite amplification and upregulation of Her2, indicating downstream activation of the pathway (Fig. 8E–F). The PI3K/mTOR pathway is frequently activated in endocrine-resistant BC and is correlated with resistance to CDK4/6 inhibitors [48, 49]. In cell lines from two models, only dual inhibition of PI3K and mTOR (by BEZ235 or gedatolisib) resulted in suppression of cell growth at low nM concentrations (Fig. 8G–H), while inhibition of PI3K (by alpelisib or buparlisib) or mTOR alone (by rapamycin) had no effect (Fig. 8I–K), likely due to feedback loops activated when only one pathway node is inhibited. Both cell lines were also sensitive to the MEK inhibitor trametinib (Fig. 8L), indicating that the MAPK pathway may play a pivotal role in cell proliferation in these models.

We confirmed that pAKT and pS6 expression were increased in B1/P/Rb_f_ and P/Rb_f_ mammary tumors and pS6 was increased in B1/P tumors compared to normal mammary gland tissue (Fig. 8M). Short-term treatment of B1/P/Rb_f_ cells with BEZ235 effectively inhibited phosphorylation of AKT and S6 (Fig. 8N). However, while short-term treatment with trametinib inhibited pERK in B1/P/Rb_f_ cells, pMEK was concomitantly increased (Fig. 8N), indicating that efficacy might be limited due to incomplete inhibition of the pathway. Taken together, these data point to the potential for overcoming resistance to CDK4/6 inhibition via drugs targeting alternate signaling pathways, and to the utility of the mammary cancer models presented here for evaluation of therapeutic combinations that target these pathways.

## Discussion

Between 10 and 20% of breast cancers arising in BRCA1 mutation carriers are ER + , and evidence of both loss of heterozygosity and homologous recombination deficiency has been found in these tumors [50]. Hormone receptor positivity may even indicate a less favorable prognosis for young women, with a higher risk of distant relapses [51], therefore a tumor model for BRCA1 loss of function together with hormone receptor positivity is relevant for evaluating new treatment strategies. Here, we describe the development and characterization of novel genetically engineered mouse mammary cancer models representing the major histological subtype of BC, the ductal carcinoma. We observed segregation into two molecular subtypes based on induced genetic aberrations. Tumors expressing part of the large T antigen (T121), and thus inhibiting proteins of the Rb family, displayed the luminal subtype of breast cancer, and cells were positive for expression of hormone receptors and Her2, regardless of their *Brca1* status, while tumors with loss of *Trp53* and *Brca1* but without Rb_f_ suppression exhibited the triple-negative phenotype.

Induction of genetic events by intraductal adeno-Cre injection into adult glands bypasses dependence on promoters that may express very early in mouse mammary development in progenitor cells, or that require lactation prior to tumor development. It also avoids development of the lymphomas that were reported *for MMTV-Cre, Rb*^*fl/fl*^*, p53*^*fl/fl*^ mice [52]. The effects of loss of Trp53, Rb and Brca1function and the resulting molecular subtype of the tumor may be critically dependent on which progenitor mammary cells are induced. For example, previously reported mouse models incorporated similar genetic aberrations using *MMTV-Cre* and *WAP-Cre* lines but exhibited oncogenesis in different subpopulations of cells within the mammary stem cell hierarchy [52, 53]. MMTV promoter-driven deletion of Rb1, p107, and Trp53 was observed to result in a mixture of luminal and EMT/spindle-like tumors [52]. Similarly, *MMTV-Cre*- and *WAP-Cre*-driven deletion of *Rb* and *Trp53* in luminal or basal primary mammary epithelial cells ex vivo gave rise to spindle cell tumors [54] that were not observed in our P/Rb_f_ model, which retained a luminal B profile and HR + status.

There are two variables that likely contribute to the differences in our results compared to previous work. First is the extent of Rb suppression, namely all three proteins of the Rb family (pRb, p107 and p130) are suppressed in our models instead of pRb alone, as in Jones et al. [54]. Suppression of Rb activity, including functions of p107 and p130 that partly overlap with pRb [55], may affect expression of an alternative set of genes than *Rb1* loss alone (supported by our RNAseq data). Second, we utilized a different induction method than in previous studies; for example, Kumar et al. [13] used *WAP-Cre* directed expression of *MMTV-T121* to suppress Rb_f_, and when combined with Trp53 and Brca1 inactivation mice developed carcinosarcomas that variably expressed basal/myoepithelial lineage markers (Keratins-5, -14) and epithelial-to-mesenchymal transition (EMT) in addition to adenocarcinoma tumors*.* In our models, suppression of Rb_f_, via *K18-T121*, combined with loss of *Brca1 and Trp53,* leads solely to development of adenocarcinomas. Thus, *MMTV-T121* combined with *WAP-Cre* induction may differ from adeno-Cre mammary intraductal injection in the nature of the mammary epithelial cells that are targeted. Additionally, parity is necessary for WAP-Cre expression, whereas our intraductal injection of adeno-Cre virus occurred in virgin glands. In summary, the histological spectrum of tumors combining loss of *Trp53* and *Brca1* with Rb inhibition is likely dependent on a combination of two parameters, the extent of Rb suppression and the particular subpopulation of mammary cells in which the induction of these aberrations occurs.

It has been proposed that the two types of mature luminal cells, ER + PR + and ER-PR-, develop from separate progenitor cells and that these two lineages are independently maintained [56]. Thus, genetic aberrations induced by WAP-Cre may be initiated in ER-PR- luminal progenitors of alveolar cells rather than in ER + PR + luminal progenitors. Based on the observed ER + PR + hyperplasia and MIN in B1/P mice, and subsequent progression to ER-PR- tumors, we propose that basal-like and luminal BC in our models may arise from a common ER + luminal progenitor population. In our models expressing the *T121* transgene, early Rb_f_ inhibition prevents progression to the ER- basal-like subtype. Whether a common luminal progenitor cell gives rise to both ER + PR + and ER-PR- cell types in normal mammary gland development is not yet determined, but cellular plasticity is a known feature of oncogenic transformation in mammary epithelial cells, and can result in conversion of luminal cells to basal-like tumors [56].

**Fig. 9:**
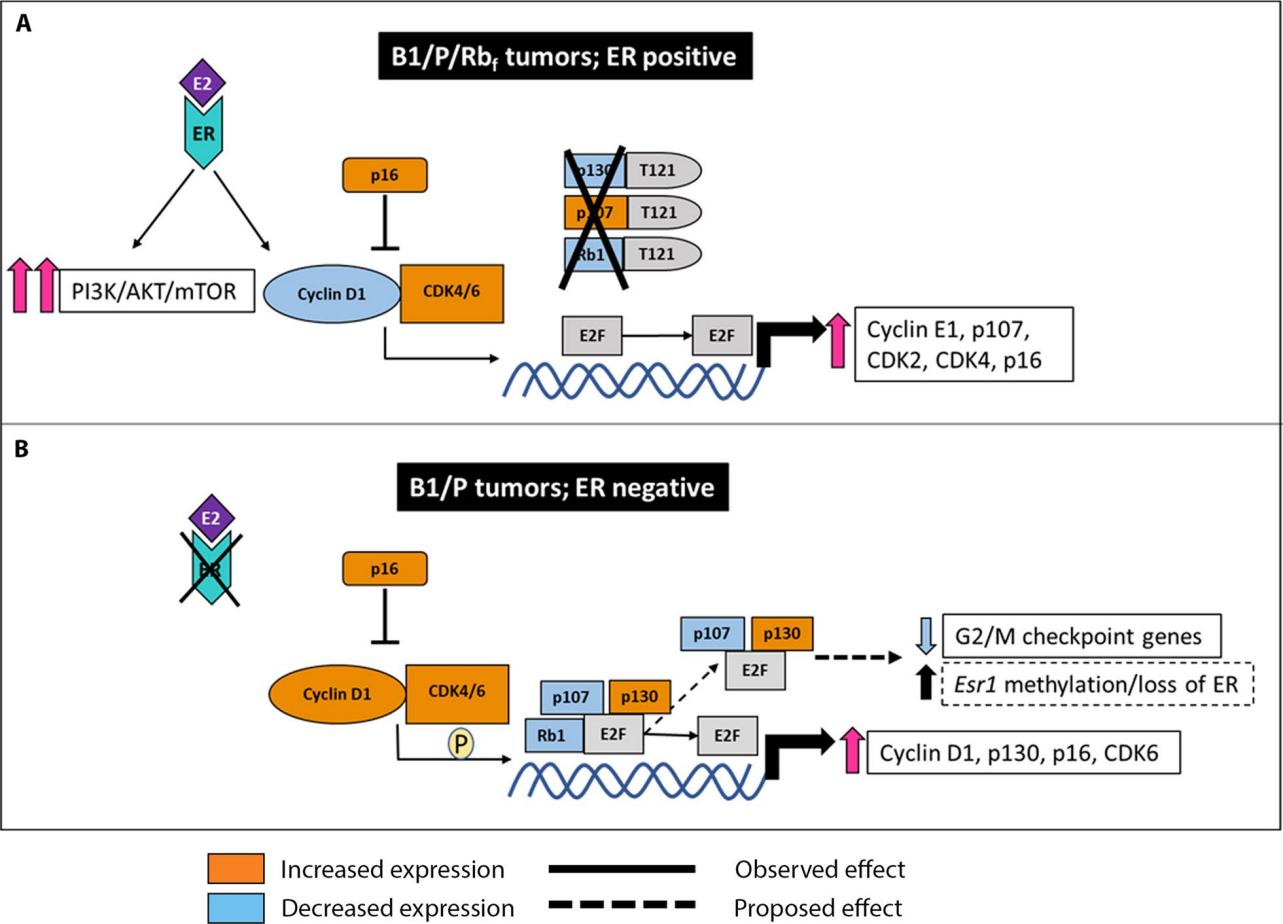
Proposed model for differences observed in the Rb pathway in ER + and ER- tumors. The blue and orange colors of the Rb pathway members indicate increased or decreased expression relative to normal mammary gland derived from RNAseq data. Black framed white boxes include observed and white boxes framed in dashed frames contain proposed outcomes of Rb pathway status. **A** In B1/P/Rb_f_ ER-positive tumors, binding of T121 suppresses the activity of pRb, p107 and p130 proteins, releasing E2F inhibition and allowing progression of transcription of multiple genes involved in cell cycle regulation, including increased expression of cyclin E1, p107, CDK2 and CDK4. Inhibition of p130 activity in particular may result in release of a p130 complex from the *Esr1* promoter that can lead to decrease in promoter methylation and thus to increased expression of ER. High ER levels, although not contributing to tumor proliferation directly via ER pathway, can still cross-activate additional pathways, such as PI3K/AKT/mTOR that were upregulated in tumors. **B** B1/P basal-like ER- tumors downregulated *Rb1.* Loss of pRb leads to release of E2F and to transcription of cell cycle regulating genes, including increased expression of cyclin D1, p130, and p16. Additional G2/M checkpoint genes can still be repressed by various complexes that p130 and p107 form with E2F proteins. Increased expression of p130 may lead to *Esr1* promoter methylation through a p130 complex on *Esr1* promoter, and thus to loss of ER expression

Our results also suggest a possible relationship between ER and the Rb pathway that determines the expression of ER in T121-expressing tumors. Previously, the presence of a p130 (*Rbl2)* multimolecular complex on the estrogen receptor alpha (*Esr1*) promoter was strongly correlated with the methylation status of *Esr1* [57, 58]. The observed increased expression of p130 in our triple-negative model could lead, through this complex, to increased methylation of *Esr1* and thus to the observed ER-negative status. Conversely, decreased levels of p130 in B1/P/Rb_f_ tumors may lead to decreased *Esr1* promoter methylation and thus to increased expression of ER that was observed in this group of tumors. However, the interactions between ER and Rb pathways are not yet fully understood, and new roles of p107 and p130 in transcriptional control of various genes are still emerging [59]. For example, members of the E2F protein family, together with Rb family proteins, can form multiple complexes with different functions in controlling downstream transcription of cell cycle genes [59]. As p130 and p107 can form distinct complexes to regulate E2F promoter motifs [59], it follows that functional loss of all three pocket proteins in B1/P/Rb_f_ tumors results in a different expression profile than *Rb1* loss (Additional file 15: Table 6) in B1/P tumors (See Fig. 9A–B diagrams). We propose that de-repression of cell cycle genes as well as *Esr1* promoter repression differ in HR + and HR- tumor models due to the change in Rb_f_ status (Fig. 9).

The HR + luminal tumor models may represent BC that is refractory or resistant to endocrine therapy. Despite the availability of newer therapeutics to treat hormone-positive breast cancers, development of resistance remains a significant problem. Over 75% of all BC fall into the Luminal A or B HR + subtypes [60] and are usually selected to receive some form of endocrine therapy such as selective estrogen receptor modulators, selective estrogen receptor down regulators or aromatase inhibitors. However, some of these patients are either nonresponsive to endocrine treatment or respond initially but later develop resistance. Interestingly, less than 10% of patients exhibit loss of ER in recurrent tumors [61], confirming that mechanisms other than lack of ER expression may be responsible for resistance to endocrine therapy. Endocrine resistance in patients has been attributed to complex pathway alterations, including activation or dysregulation of the PI3K/AKT/mTOR, RAF/MEK/ERK and Rb pathways [62, 63]. The cyclin D1-CDK4/6-INK4-RB pathway is the key regulator of the G1-S transition of the cell cycle [49] and increased expression of cyclin D1, phosphorylation of RB protein though CDK4/6/cyclin D1, and increased dissociation of RB from the E2F transcription factor are associated with emergence of endocrine resistance and warrant the use of CDK inhibitors [49]. Therapeutic approaches similar to those that have been successful in HR- subtypes may be required for the treatment of CDK4/6 inhibitor-resistant patients who have lost sensitivity to hormone expression. The PI3K/AKT/mTOR signaling pathway is activated in approximately 30–40% of BC, particularly in the HR + subtype [49]. AKT activation was previously found to be significantly associated with resistance to endocrine therapy in patients with metastatic breast cancer [64]. Furthermore, correlation of the PIK3/AKT/mTOR pathway with resistance to CDK4/6 inhibitors has also been reported [49]. The models presented here exhibit upregulation of PI3K signaling and sensitivity to pathway inhibition, presenting a platform for evaluation of combined targeted therapies to improve chemotherapeutic response in patients. Multiple mechanisms of resistance to CDK4/6 inhibitors activate the MAPK pathway in HR + metastatic breast cancer cells [65], thus the sensitivity of our model to MEK inhibition may be extended to additional agents that target the MAPK pathway.

Temporal control of induction allows for initiation of tumorigenesis in the fully developed mammary gland without the need for lactation, which recapitulates progression of cancer in patients more accurately. Thus, these mammary tumor models allow for the evaluation of cancer prevention strategies, especially targeted toward *Brca1* mutation carriers. Treatment with therapeutic candidates may be implemented as early as two weeks post-induction, prior to detection of the earliest lesions. One drawback of adeno-Cre mediated induction in the gland is potential variation in transduction efficiency throughout the ductal epithelium. Additional heterogeneity could be introduced by unequal levels of recombination of different floxed alleles. Sufficient cohort sizes must be employed in prevention studies to account for such variations and to allow for statistically significant results. However, for tumor growth inhibition studies, the orthotopic allograft model is a tractable platform in which recombination can be validated, while recapitulating the GEM histopathology features desired for translational research. (Additional file 16: Original uncropped Western blots; Additional file 17: Supplementary Methods).

## Conclusions

The mammary cancer models presented here recapitulate luminal and basal-like BC subtypes in patients based on histopathology features and gene expression profiles. Tumors of both B1/P/Rb_f_ and P/Rb_f_ HR-positive luminal models represent CDK4/6 inhibitor-resistant and endocrine therapy resistant BC. Together with the strain-matched B1/P HR-negative model, these mouse models are a valuable tool for exploration of combined targeted therapies for both types of drug resistance, as well as a platform for exploration of resistance mechanisms.

## Supplementary Information

**Additional file 1. Table S1.** List of antibodies and staining conditions that were used for immunohistochemistry.

**Additional file 2. Fig. S1.** Recombination of floxed alleles in tumors was confirmed by PCR specific for each recombined allele. **A** PCR results for recombination of *Trp53*, *TgK18GT*_*121*_ and *Brca1* in B1/P/Rb_f_ tumors. **B** PCR results for recombination of *Trp53*, and *Brca1* in B1/P tumors. **C** PCR results for recombination of *Trp53* and *TgK18GT*_*121*_ in P/Rb_f_ tumors. **D** Results of RT-qPCR for *Brca1* expression in mammary tumors that were used for RNAseq analysis. The expression was normalized to a P/Rbf tumor with wild type *Brca1* expression (#287845). Results confirm loss of Brca1 expression in all samples except #208863 where partial loss was observed.

**Additional file 3. Fig. S2.** Comparison of receptor expression in HR-positive and HR-negative tumors. Results from quantitative RT-PCR for ER (*Esr1*) and PR (*Pgr*) in B1/P/Rb_f_(*N*=5) and B1/P (*N*=3) mammary tumors indicate lack of expression for both receptors in B1/P tumors. Beta-actin was used as an internal control. Comparative Ct method was used to evaluate the relative quantity of the target genes using 2^-deltaCt^ method, where delta Ct= mean Ct_target_ gene -C_tactin_. ** indicates *p* < 0.01 by unpaired *t*-test.

**Additional file 4. Fig. S3.** Expression of basal marker p63 in mammary tumors of different genotypes. B1/P/Rb_f_ and P/Rb_f_ tumors exhibited few randomly scattered p63 positive cells, confirming categorization of tumors as adenocarcinomas of no specific type. B1/P tumors exhibited a variety of staining patterns with dense populations of positive cells, however, the histology was consistent with adenocarcinoma, not adenomyoepithelioma. Examples of p63 staining in tumors with squamous differentiation (asterisk) and mesenchymal differentiation is also shown. Scale bar 200µm.

**Additional file 5. Fig. S4.** B1/P/Rb_f_ tumors show amplification of *Erbb2* gene (Her2). FISH for murine *Erbb2* (green signal) detects an additional copy on chromosome 14 (labelled in orange) besides the 2 endogenous copies on chromosomes 11 (unlabeled).

**Additional file 6. Fig. S5.** Orthotopic allograft tumors recapitulate marker expression in GEM tumors. IHC analysis of molecular markers in mammary allograft tumors of B1/P/Rb_f_ (**A**) and B1/P (**B**) genotypes. ER=estrogen receptor, PR=progesterone receptor, Her2= human epidermal growth factor receptor 2, CK14=cytokeratin 14, CK18=cytokeratin 18, VIM=vimentin, Ki67= marker of cell proliferation Ki-67. Brown (DAB) or red stain (Nova red) indicate positive staining. Scale bar 200µm

**Additional file 7. Fig. S6.** Comparison of allograft models generated by intraductal versus mammary fat pad injection of cancer cells. **A**) IHC for estrogen (ER) and progesterone receptors (PR) show no major difference in expression in B1/P/Rbf tumors. Scale bar 200µm. **B**) Fat pad and intraductal injections generate allografts with comparable latency (shown in weeks)

**Additional file 8. Fig. S7.** RNAseq data analysis. **A**) Hierarchical clustering of normalized RNAseq data for the top 300 genes filtered by variance shows two distinct signatures in tumors. **B**) GSEA comparing B1/P and B1/P/Rbf tumors revealed significant differences in G2M checkpoint genes.

**Additional file 9. Table S2.** Differential expression of genes (DEG) analysis among tumor models. DEG was performed by applying Limma Voom package at p value threshold set to 0.001. Data are ordered by logFC values.

**Additional file 10. Table S3.** Gene Set Enrichment Analysis (GSEA) results for all models. GSEA against a ranked set of genes (MSigDB v6.2 Human/Mouse/Macaque) was performed for each contrast using C2 curated gene sets. Only significantly enriched gene sets are listed (*p* adj <0.05). Gene sets for which heat maps are shown in figures are highlighted in yellow.

**Additional file 11. Fig. S8.** IPA diagrams show aberrations in the Rb pathway in both B1/P/Rb_f_ (**A**) and B1/P (**B**) tumors. Purple rectangles highlight the major differences in Rb pathway gene expression betweenthe two tumor models. Data were analyzed through the use of Ingenuity Pathway Analysis (Bioinformatics. 2014 Feb 15;30(4):523-30).

**Additional file 12. Table S4.** Additional Gene Set Enrichment Analysis (GSEA) results with KEGG and Biocarta for the comparison of the two luminal models. GSEA against a ranked set of genes (MSigDB v6.2 Human/Mouse/Macaque) was performed for B1/P/Rb_f_compared to P/Rb_f_ tumors using BIOCARTA and KEGG curated gene sets. Only significantly enriched gene sets are listed (padj <0.05). Gene sets for which heat maps are shown in figures are highlighted in yellow

**Additional file 13. Fig. S9.** Several examples of pathways enriched in GSEA comparing B1/P/Rb_f_ and P/Rbf_f_ models. Relative enrichment was observed in cell cycle **A**, DNA replication **B** and nucleotide excision repair **C** gene sets. Differences in expression of Mcm gene family **D** were common for several of these pathways (orange rectangles in A and B). **E** Expression of genes in the Mcm family in all three tumor models and control mammary glands. Highest expression was observed in B1/P/Rb_f_ tumors.

**Additional file 14. Table S5.** List of significantly enriched canonical pathways obtained from GSEA for tumors of all three genotypes when compared to control mammary glands. Listed pathways were used in single-sample GSEA (ssGSEA) on all samples.

**Additional file 15. Table S6.** Differential expression of genes (DEG) analysis among tumor models. DEG was performed by applying Limma Voom package at p value threshold set to 0.05. Data are ordered by contrast and logFC values.

**Additional file 16.** Original uncropped Western blots.

**Additional file 17.** Supplementary Methods.

## Data Availability

The RNA sequencing datasets supporting the conclusions of this article are available in the NCBI Gene Expression Omnibus (GEO) repository under submission GSE206068.

## Abbreviations

B1/P/Rb_f_: Brca1^fl/fl^/Trp53^fl/fl^/TgK18GT_121_^Tg/+^
B1/P: Brca1^fl/fl^/Trp53^fl/fl^
BC: Breast cancer
BLG: Bovine *β*-lactoglobulin
Brca1: Breast cancer type 1 susceptibility
CK14: Cytokeratin 14
CK18: Cytokeratin 18
DEG: Differentially expressed genes
ER: Estrogen receptor
FISH: Fluorescence in situ hybridization
GEMM: Genetically engineered mouse model
GSEA: Gene set enrichment analysis
Her2: Human epidermal growth factor receptor 2
HR: Hormone receptor
IHC: Immunohistochemistry
MIN: Mammary intraepithelial neoplasia
MMTV: Mouse mammary tumor virus
p.i.: Post-induction
P/Rb_f_: Trp53^fl/fl^/TgK18GT_121_^Tg/+^
PR: Progesterone receptor
Rb: Retinoblastoma
T121: Part of the SV40 large T antigen (121 aa)
Trp53: Tumor protein 53
VIM: Vimentin
WAP: Whey acidic protein
