# Supplementary figures and images for: Loss of Brca1 and Trp53 in adult mouse mammary ductal epithelium results in development of hormone receptor-positive or hormone receptor-negative tumors, depending on inactivation of Rb family proteins
Source: Breast Cancer Res. 2022 Nov 4;24:75. doi: 10.1186/s13058-022-01566-4 (PMC9636824; doi:10.1186/s13058-022-01566-4)

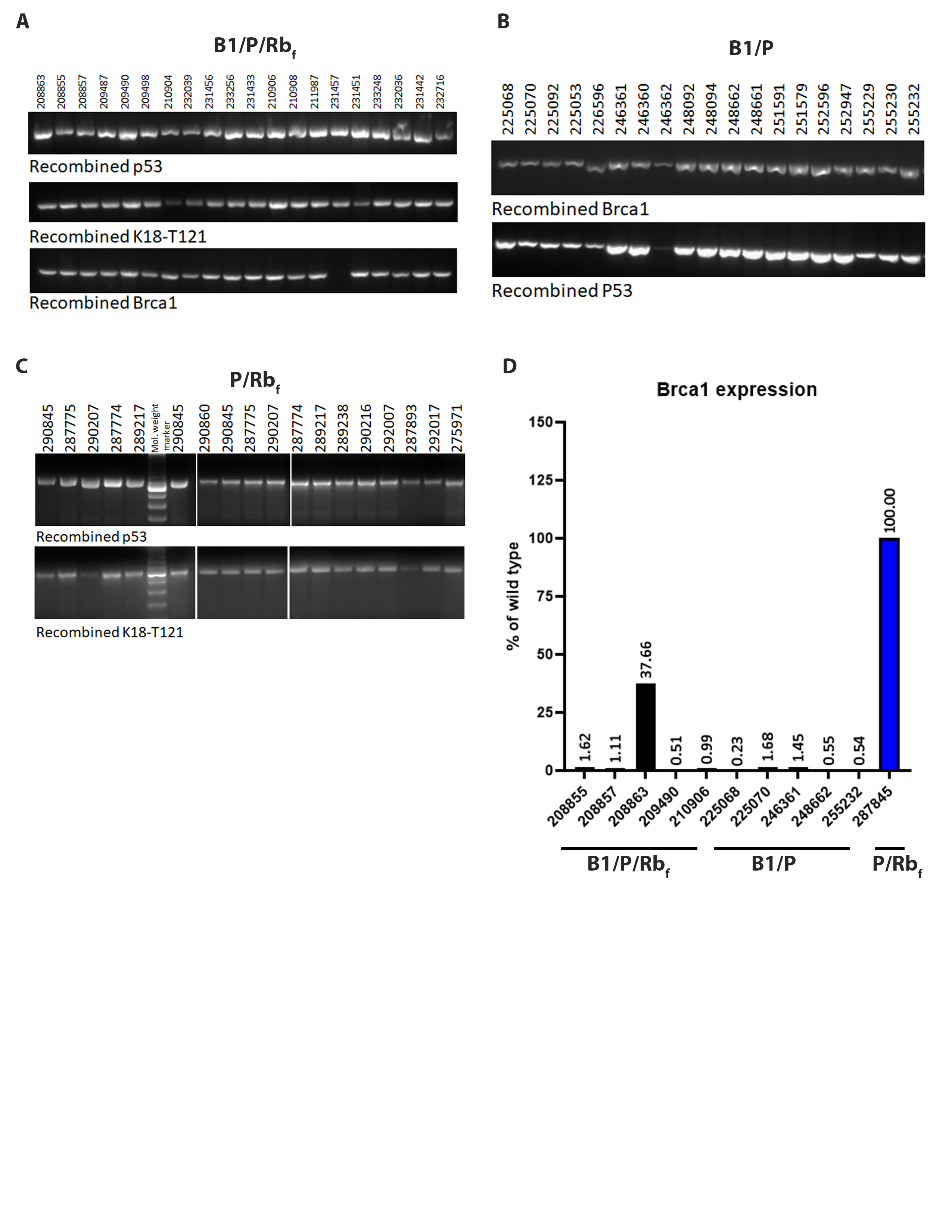

Supplement: Supplementary file 2 — Additional file 2. Fig. S1. Recombination of floxed alleles in tumors was confirmed by PCR specific for each recombined allele. A PCR results for recombination of Trp53, TgK18GT121 and Brca1 in B1/P/Rbf tumors. B PCR results for recombination of Trp53, and Brca1 in B1/P tumors. C PCR results for recombination of Trp53 and TgK18GT121 in P/Rbf tumors. D Results of RT-qPCR for Brca1 expression in mammary tumors that were used for RNAseq analysis. The expression was normalized to a P/Rbf tumor with wild type Brca1 expression (#287845). Results confirm loss of Brca1 expression in all samples except #208863 where partial loss was observed. [file 13058_2022_1566_MOESM2_ESM.jpg]

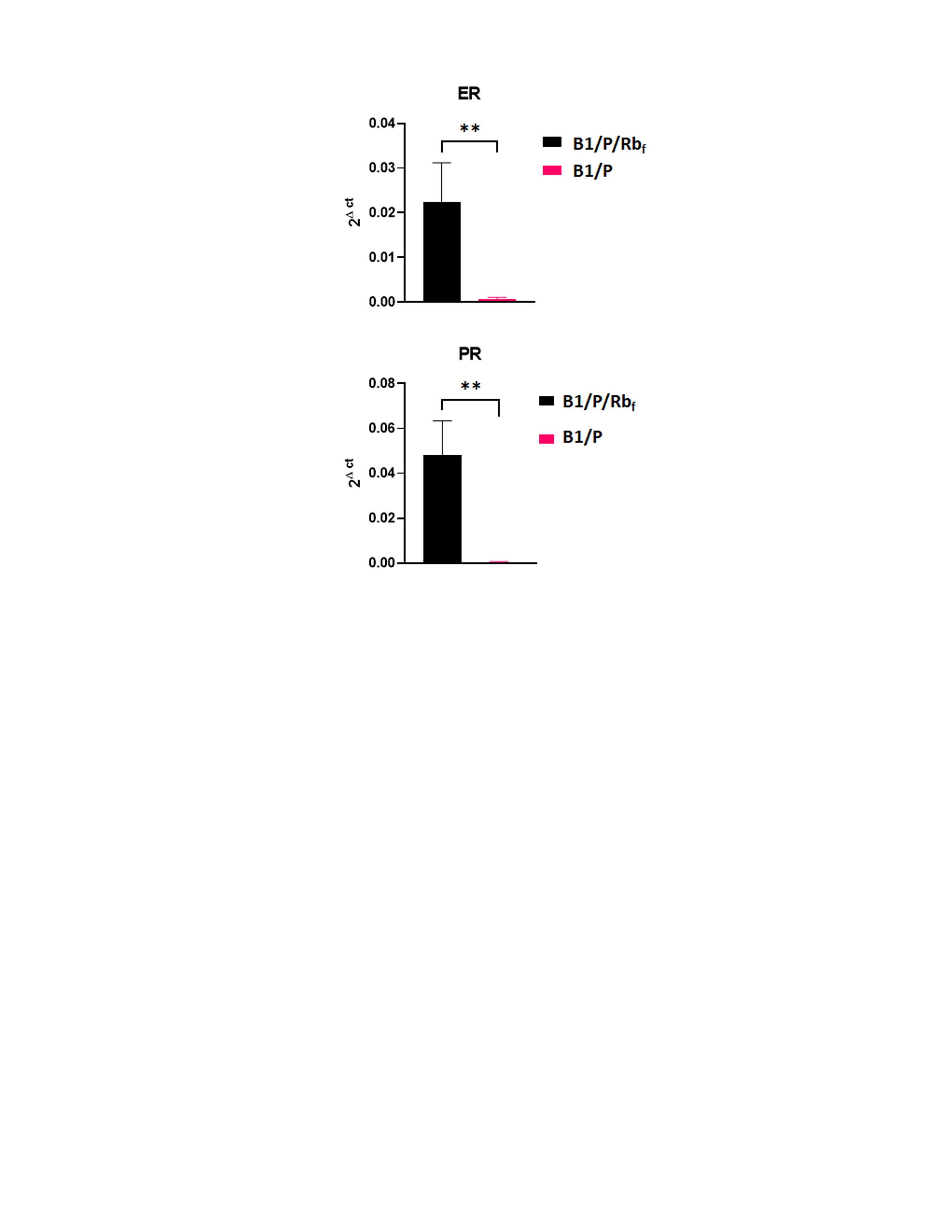

Supplement: Supplementary file 3 — Additional file 3. Fig. S2. Comparison of receptor expression in HR-positive and HR-negative tumors. Results from quantitative RT-PCR for ER (Esr1) and PR (Pgr) in B1/P/Rbf(N=5) and B1/P (N=3) mammary tumors indicate lack of expression for both receptors in B1/P tumors. Beta-actin was used as an internal control. Comparative Ct method was used to evaluate the relative quantity of the target genes using 2-deltaCt method, where delta Ct= mean Cttarget gene -Ctactin. ** indicates p < 0.01 by unpaired t-test. [file 13058_2022_1566_MOESM3_ESM.jpg]

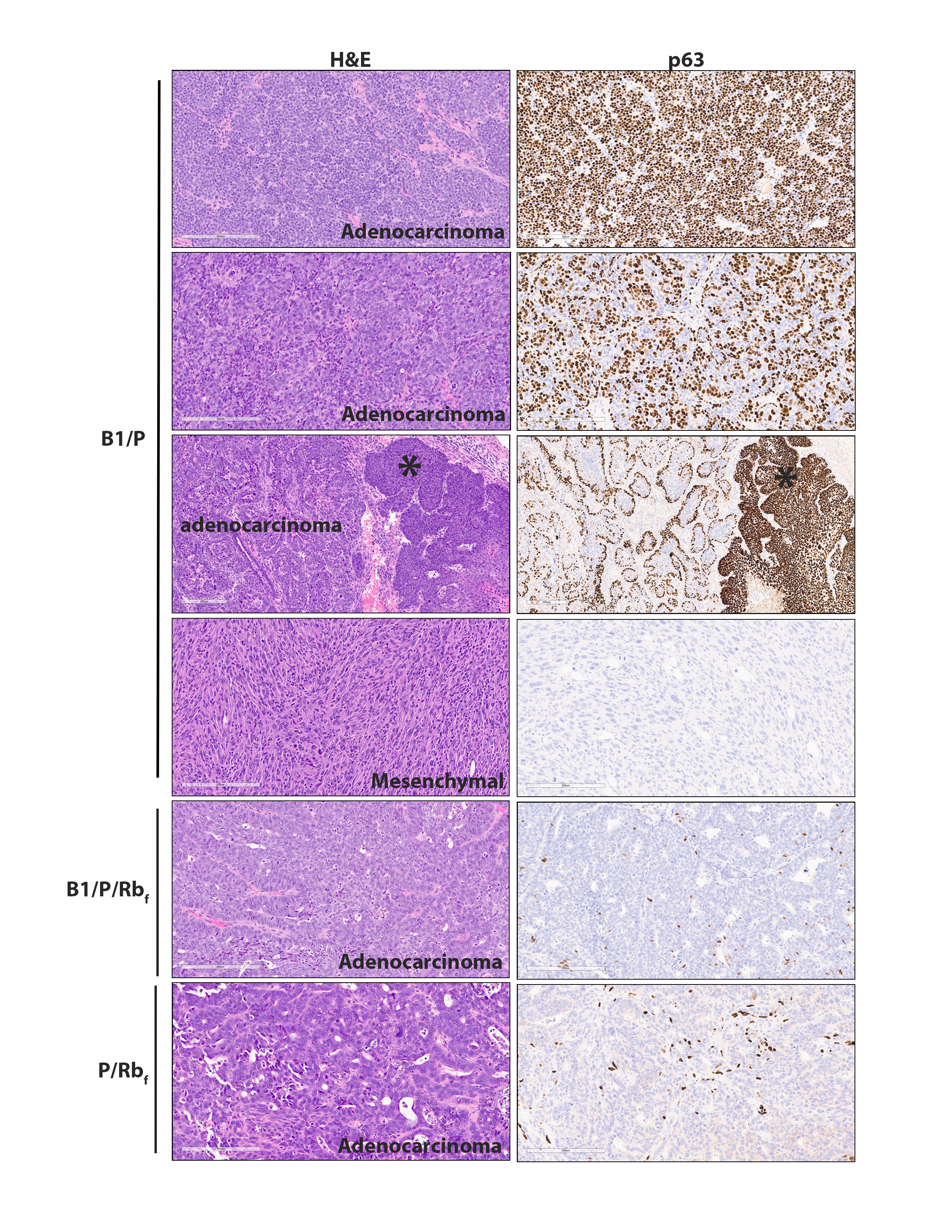

Supplement: Supplementary file 4 — Additional file 4. Fig. S3. Expression of basal marker p63 in mammary tumors of different genotypes. B1/P/Rbf and P/Rbf tumors exhibited few randomly scattered p63 positive cells, confirming categorization of tumors as adenocarcinomas of no specific type. B1/P tumors exhibited a variety of staining patterns with dense populations of positive cells, however, the histology was consistent with adenocarcinoma, not adenomyoepithelioma. Examples of p63 staining in tumors with squamous differentiation (asterisk) and mesenchymal differentiation is also shown. Scale bar 200µm. [file 13058_2022_1566_MOESM4_ESM.jpg]

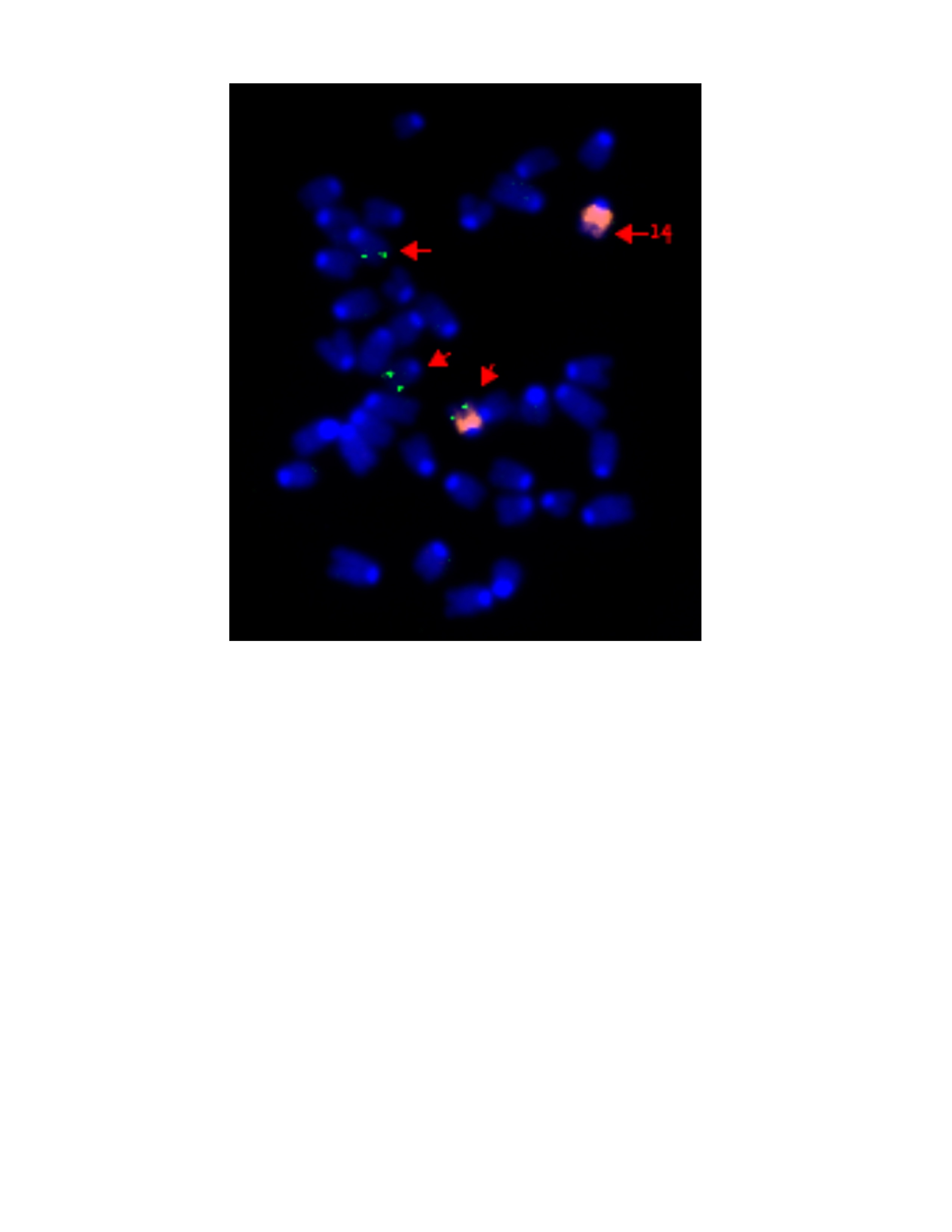

Supplement: Supplementary file 5 — Additional file 5. Fig. S4. B1/P/Rbf tumors show amplification of Erbb2 gene (Her2). FISH for murine Erbb2 (green signal) detects an additional copy on chromosome 14 (labelled in orange) besides the 2 endogenous copies on chromosomes 11 (unlabeled). [file 13058_2022_1566_MOESM5_ESM.jpg]

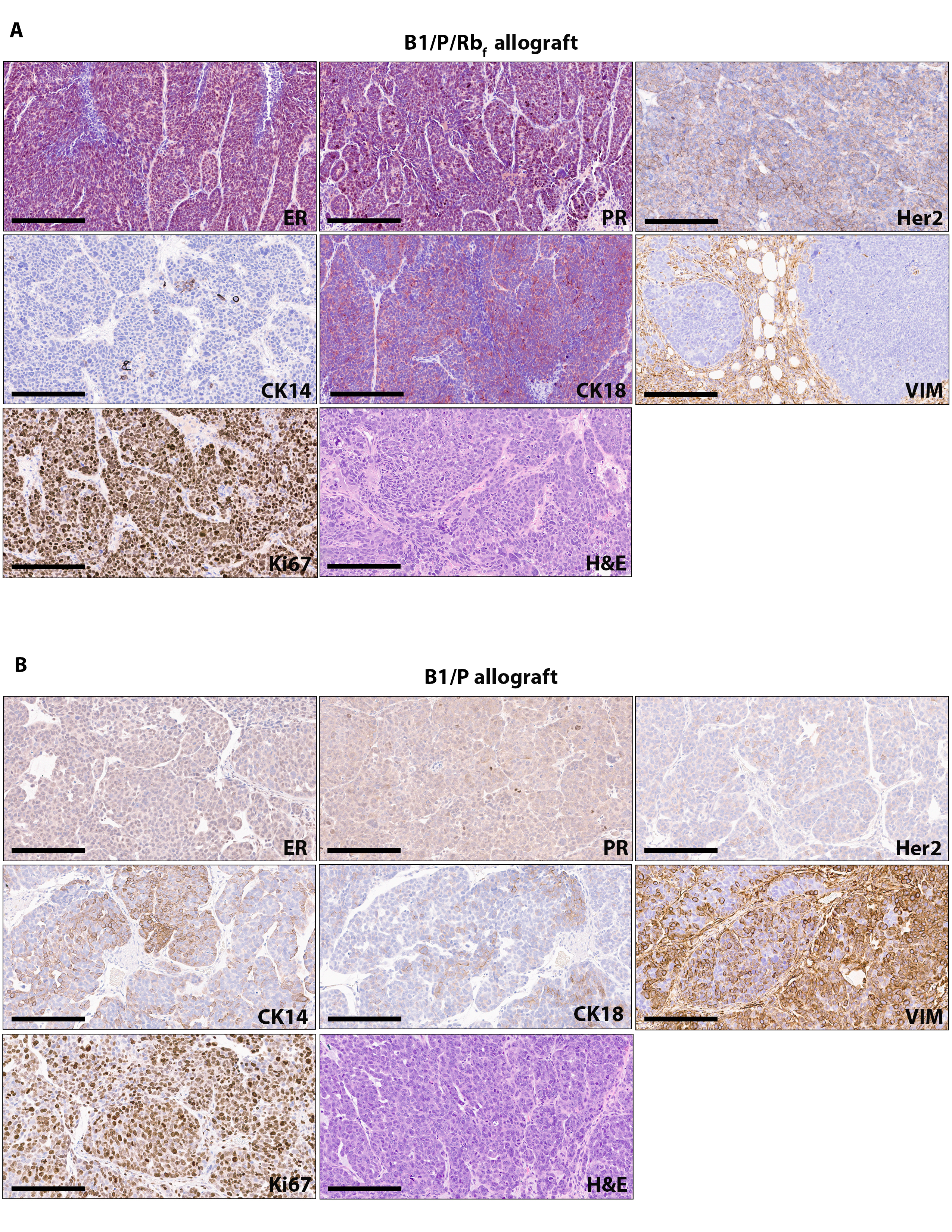

Supplement: Supplementary file 6 — Additional file 6. Fig. S5. Orthotopic allograft tumors recapitulate marker expression in GEM tumors. IHC analysis of molecular markers in mammary allograft tumors of B1/P/Rbf (A) and B1/P (B) genotypes. ER=estrogen receptor, PR=progesterone receptor, Her2= human epidermal growth factor receptor 2, CK14=cytokeratin 14, CK18=cytokeratin 18, VIM=vimentin, Ki67= marker of cell proliferation Ki-67. Brown (DAB) or red stain (Nova red) indicate positive staining. Scale bar 200µm [file 13058_2022_1566_MOESM6_ESM.jpg]

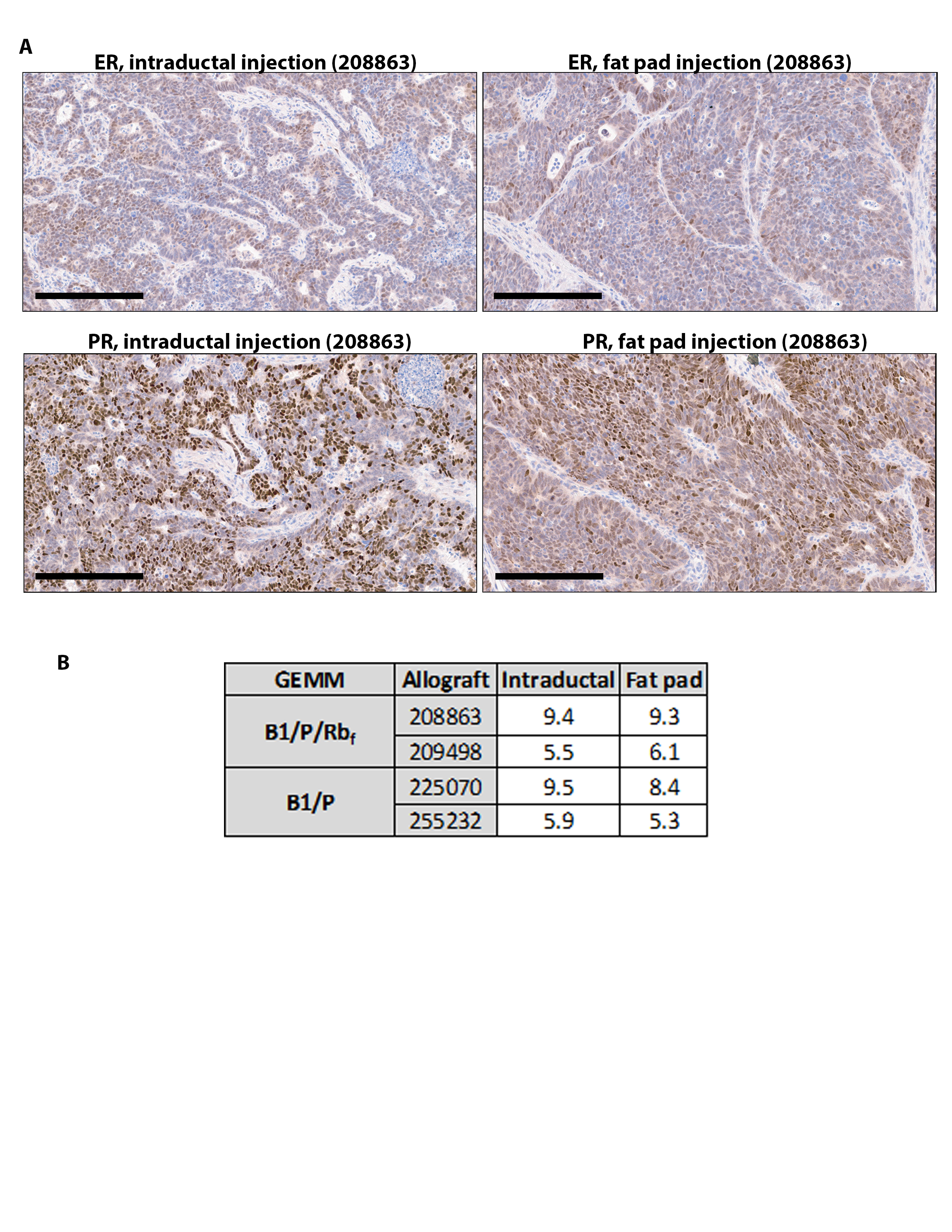

Supplement: Supplementary file 7 — Additional file 7. Fig. S6. Comparison of allograft models generated by intraductal versus mammary fat pad injection of cancer cells. A) IHC for estrogen (ER) and progesterone receptors (PR) show no major difference in expression in B1/P/Rbf tumors. Scale bar 200µm. B) Fat pad and intraductal injections generate allografts with comparable latency (shown in weeks) [file 13058_2022_1566_MOESM7_ESM.jpg]

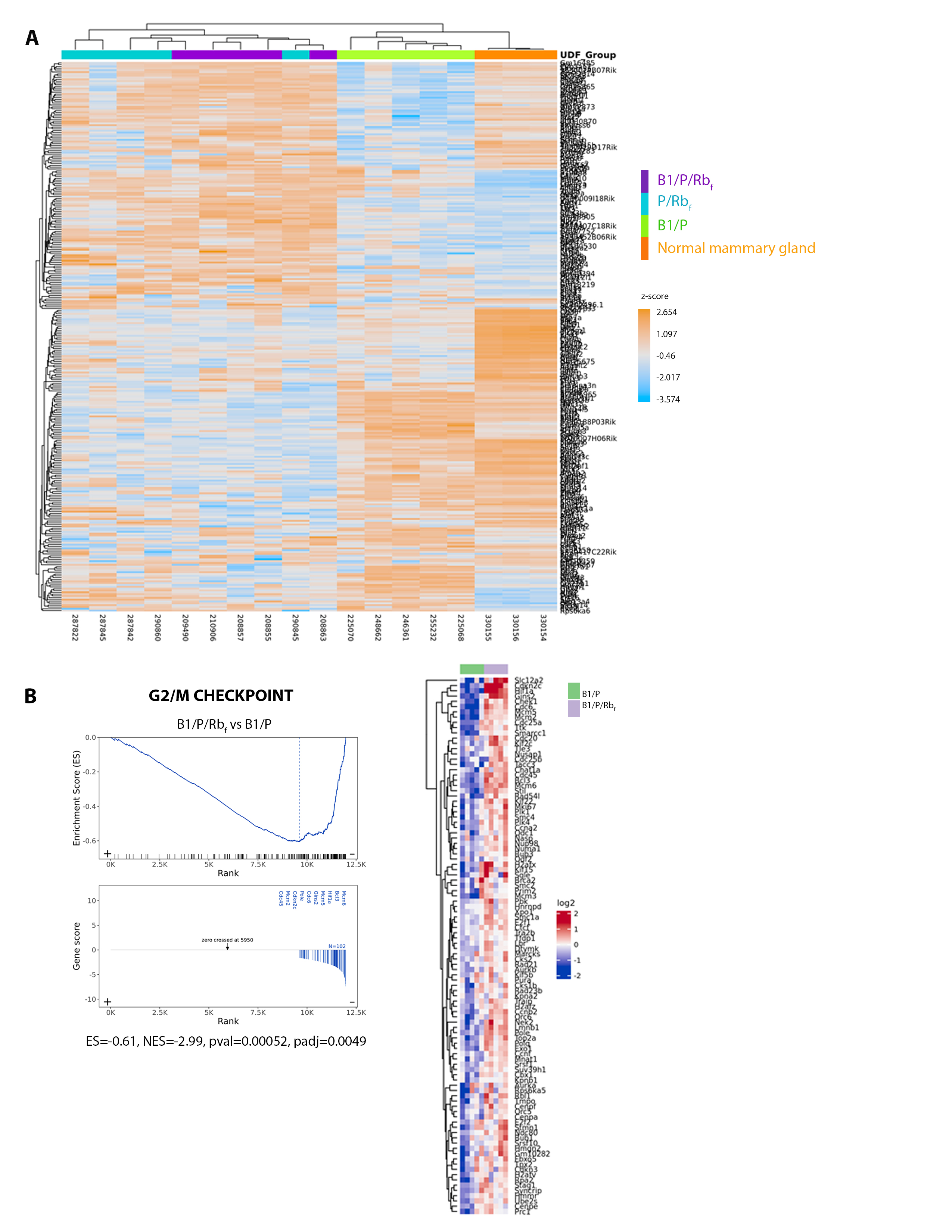

Supplement: Supplementary file 8 — Additional file 8. Fig. S7. RNAseq data analysis. A) Hierarchical clustering of normalized RNAseq data for the top 300 genes filtered by variance shows two distinct signatures in tumors. B) GSEA comparing B1/P and B1/P/Rbf tumors revealed significant differences in G2M checkpoint genes. [file 13058_2022_1566_MOESM8_ESM.jpg]

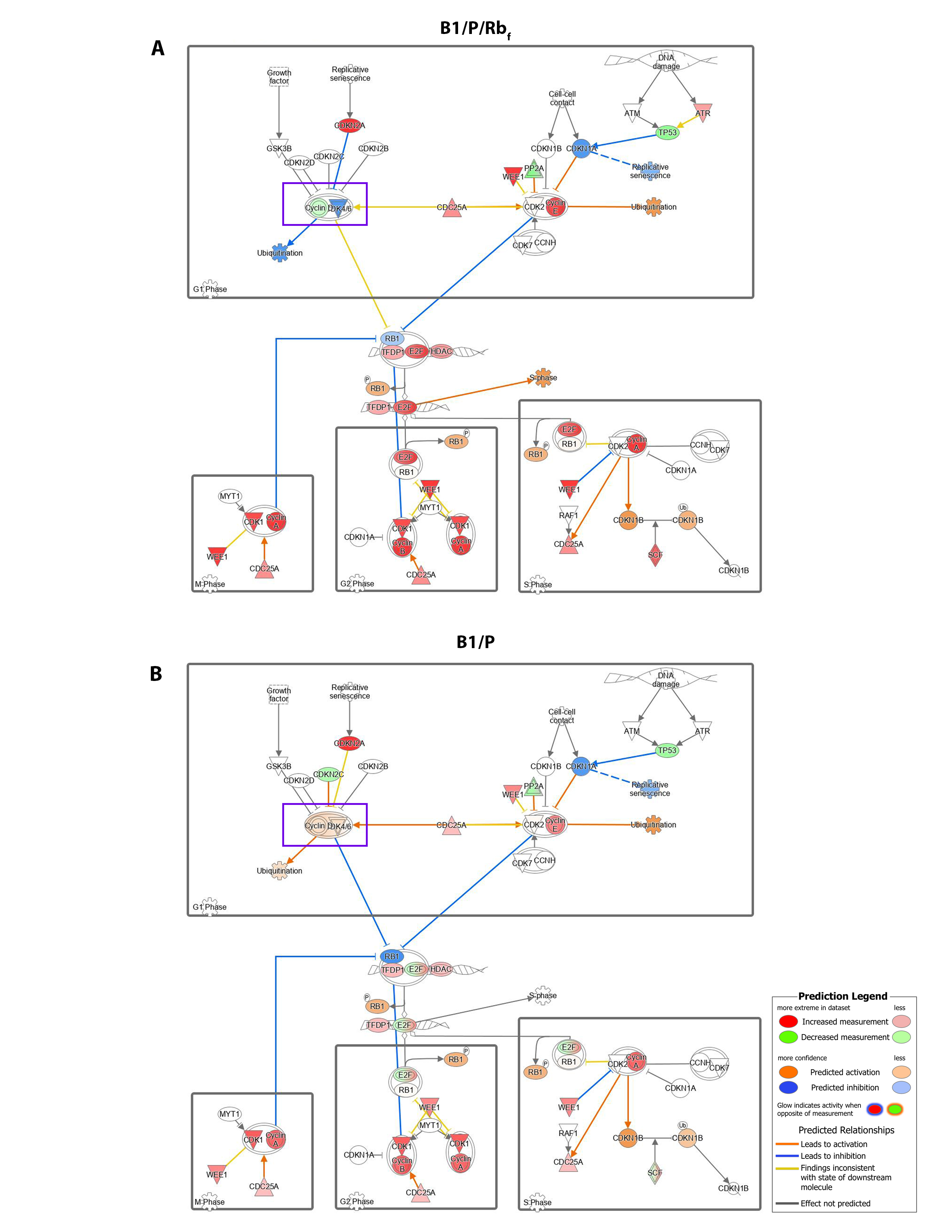

Supplement: Supplementary file 11 — Additional file 11. Fig. S8. IPA diagrams show aberrations in the Rb pathway in both B1/P/Rbf (A) and B1/P (B) tumors. Purple rectangles highlight the major differences in Rb pathway gene expression betweenthe two tumor models. Data were analyzed through the use of Ingenuity Pathway Analysis (Bioinformatics. 2014 Feb 15;30(4):523-30). [file 13058_2022_1566_MOESM11_ESM.jpg]

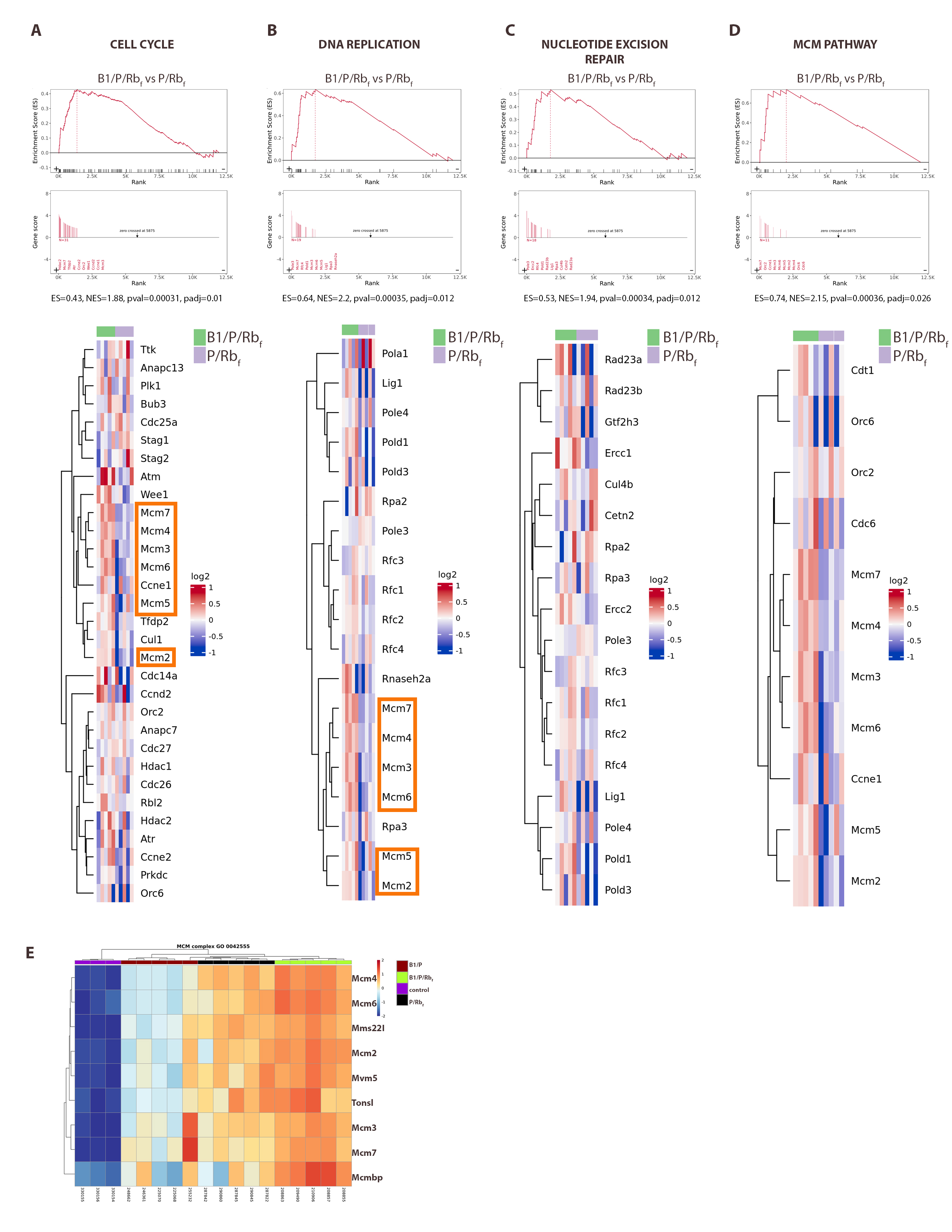

Supplement: Supplementary file 13 — Additional file 13. Fig. S9. Several examples of pathways enriched in GSEA comparing B1/P/Rbf and P/Rbff models. Relative enrichment was observed in cell cycle A, DNA replication B and nucleotide excision repair C gene sets. Differences in expression of Mcm gene family D were common for several of these pathways (orange rectangles in A and B). E Expression of genes in the Mcm family in all three tumor models and control mammary glands. Highest expression was observed in B1/P/Rbf tumors. [file 13058_2022_1566_MOESM13_ESM.jpg]
