# Supplementary material for: Loss of Brca1 and Trp53 in adult mouse mammary ductal epithelium results in development of hormone receptor-positive or hormone receptor-negative tumors, depending on inactivation of Rb family proteins
Source: Breast Cancer Res. 2022 Nov 4;24:75. doi: 10.1186/s13058-022-01566-4 (PMC9636824; doi:10.1186/s13058-022-01566-4)
Supplement: Supplementary file 16 — Additional file 16. Original uncropped Western blots. [file 13058_2022_1566_MOESM16_ESM.pptx]

## Slide 1
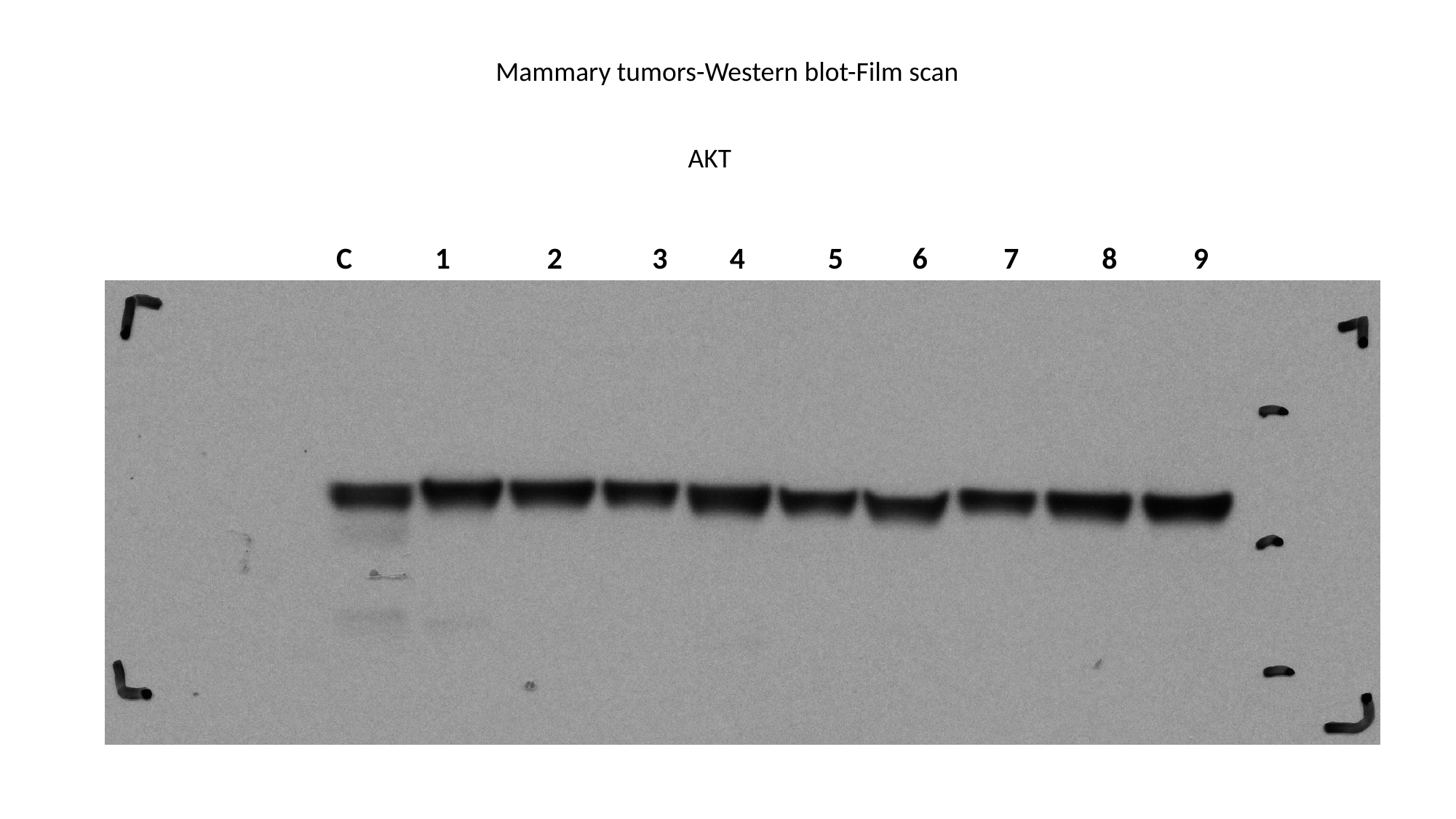

Mammary tumors-Western blot-Film scan
AKT
C 1 2 3 4 5 6 7 8 9

## Slide 2
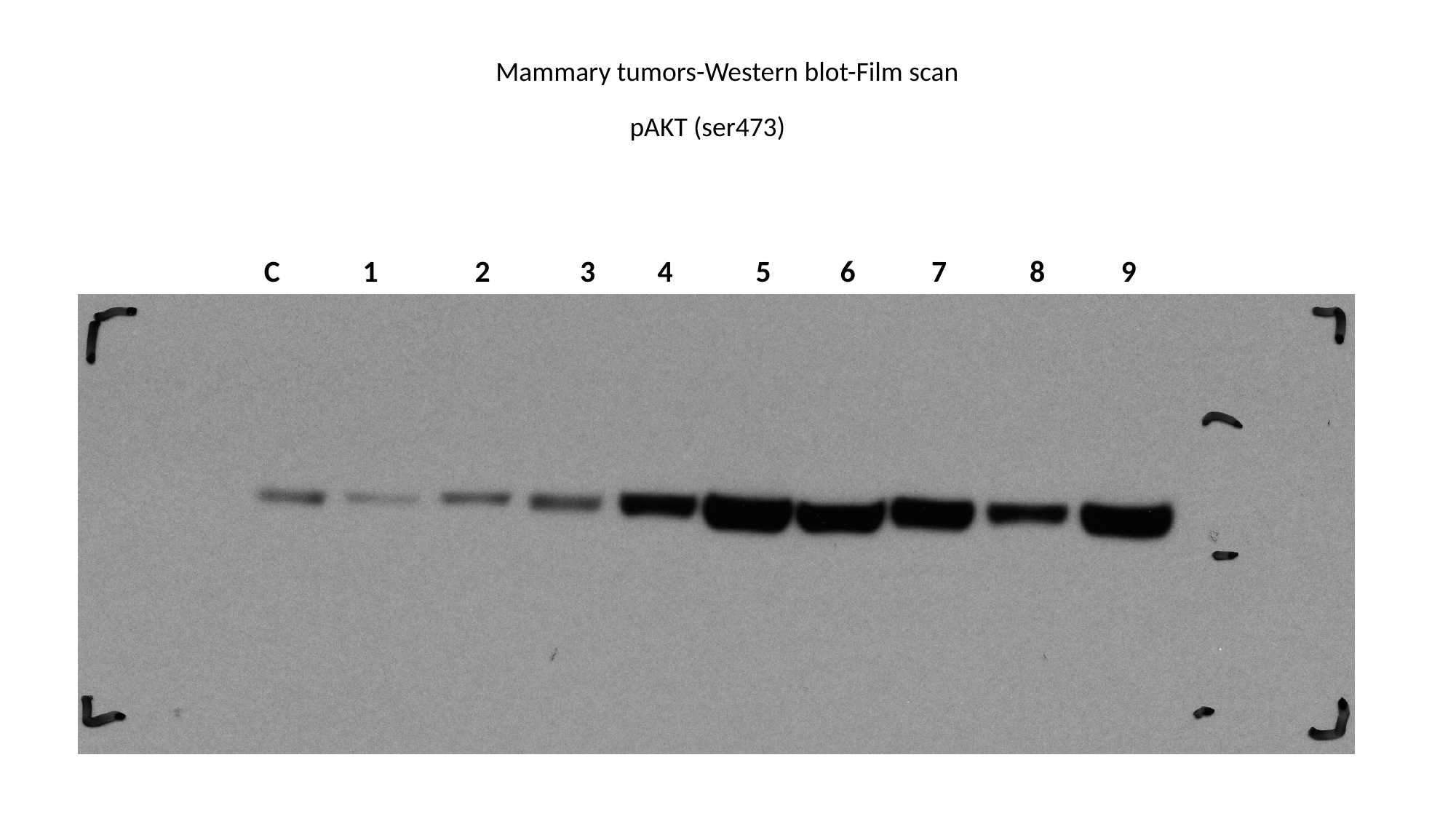

Mammary tumors-Western blot-Film scan
pAKT (ser473)
C 1 2 3 4 5 6 7 8 9

## Slide 3
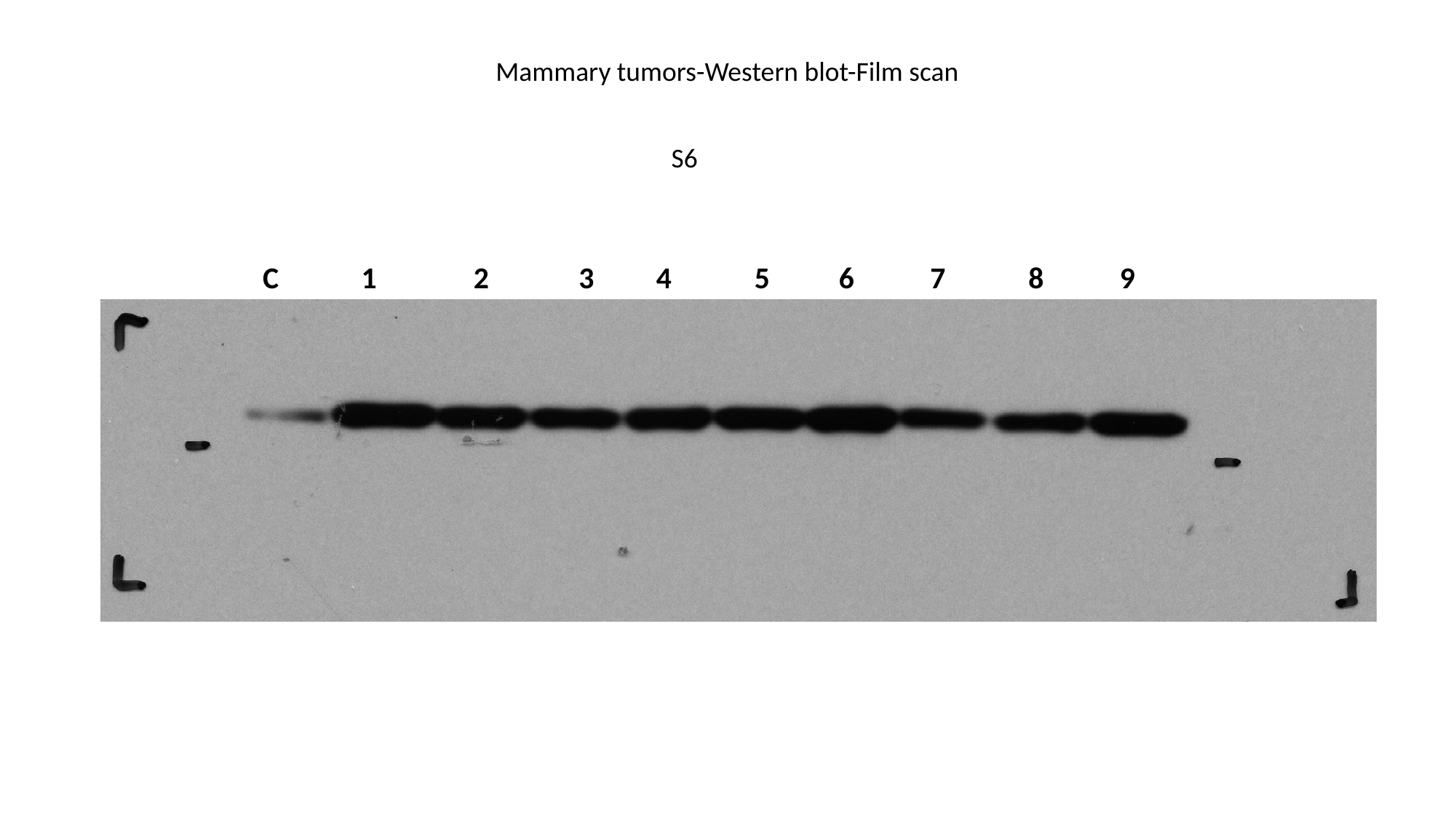

Mammary tumors-Western blot-Film scan
S6
C 1 2 3 4 5 6 7 8 9

## Slide 4
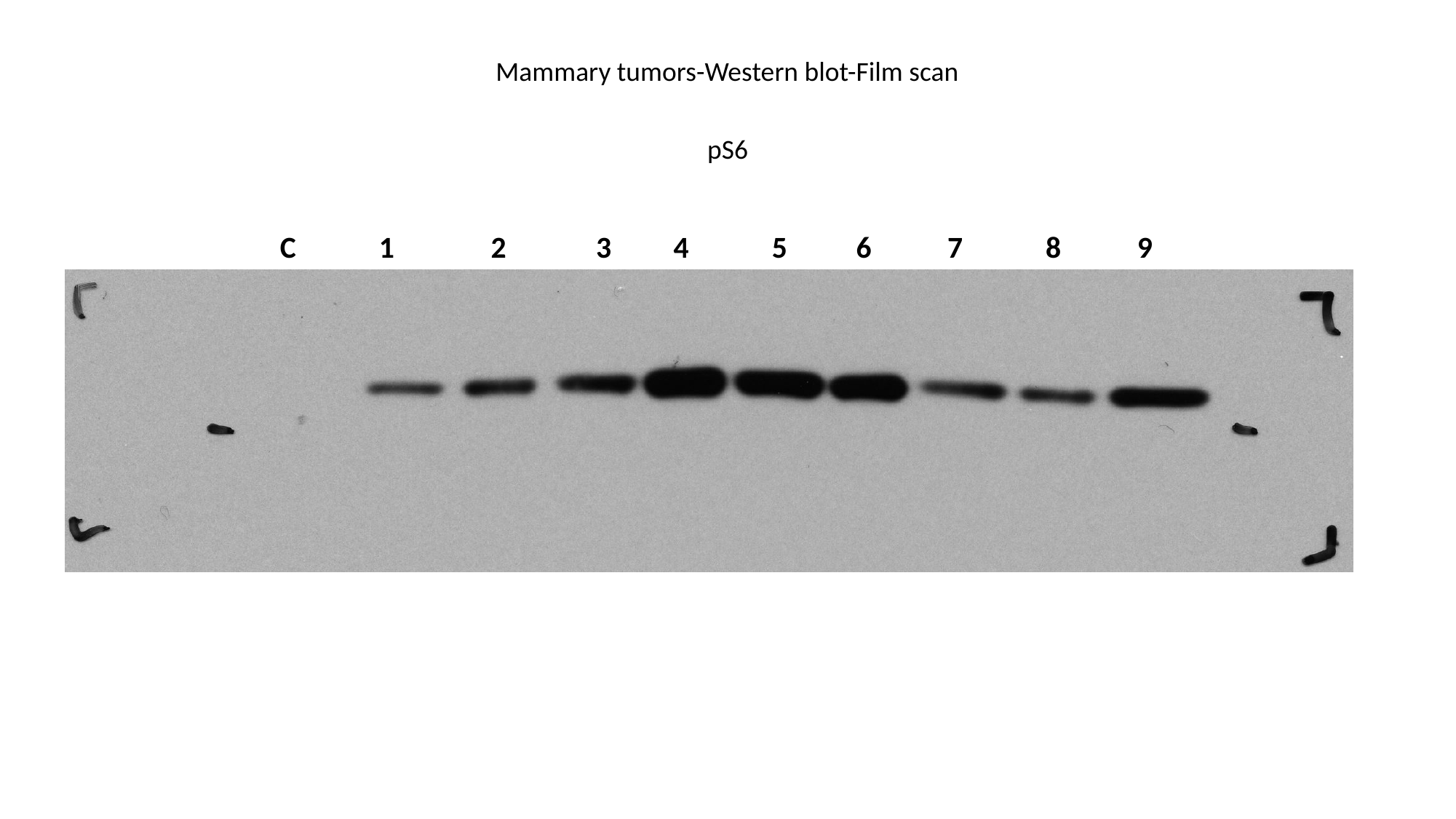

Mammary tumors-Western blot-Film scan
pS6
C 1 2 3 4 5 6 7 8 9

## Slide 5
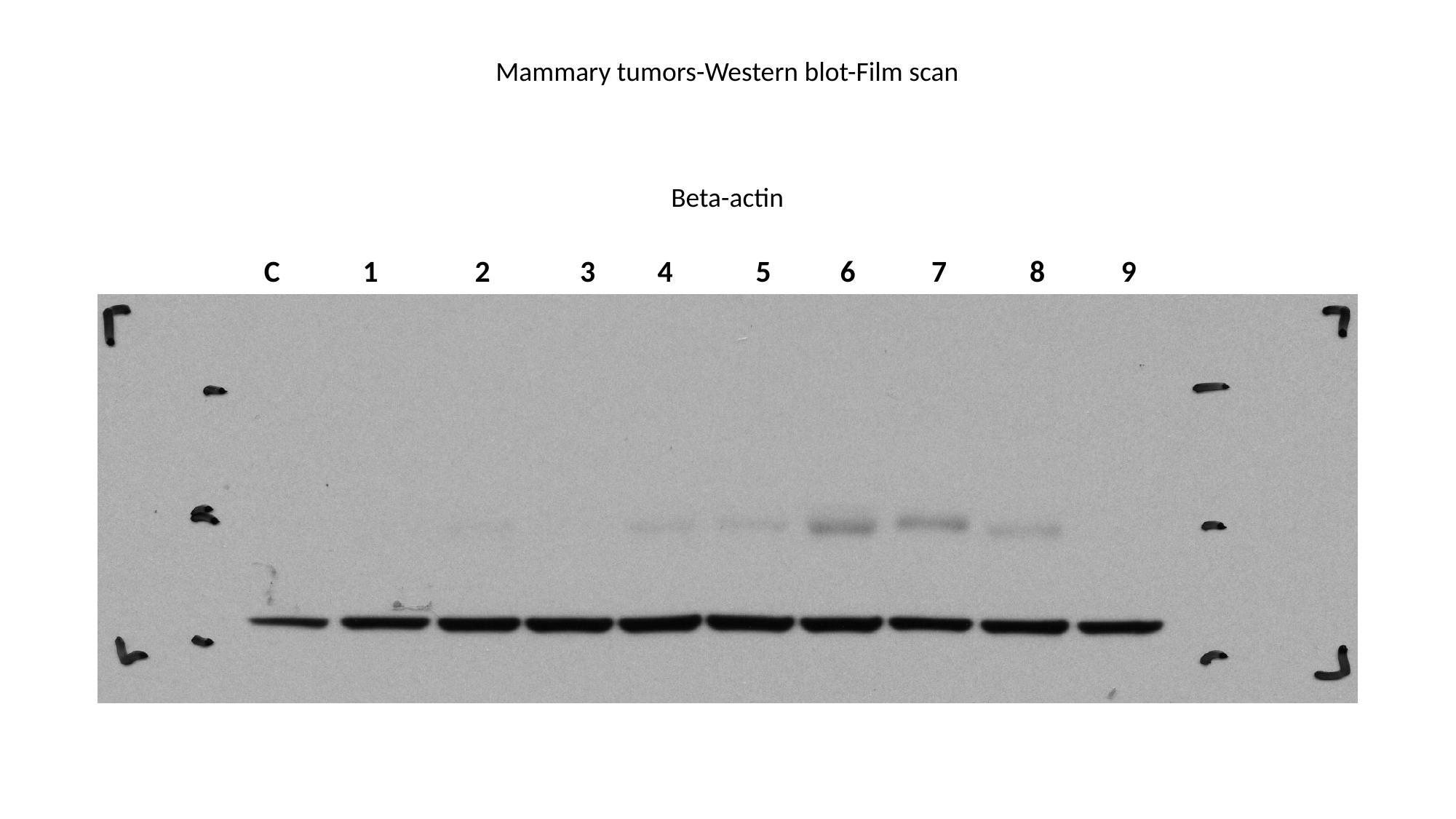

Mammary tumors-Western blot-Film scan
Beta-actin
C 1 2 3 4 5 6 7 8 9

## Slide 6
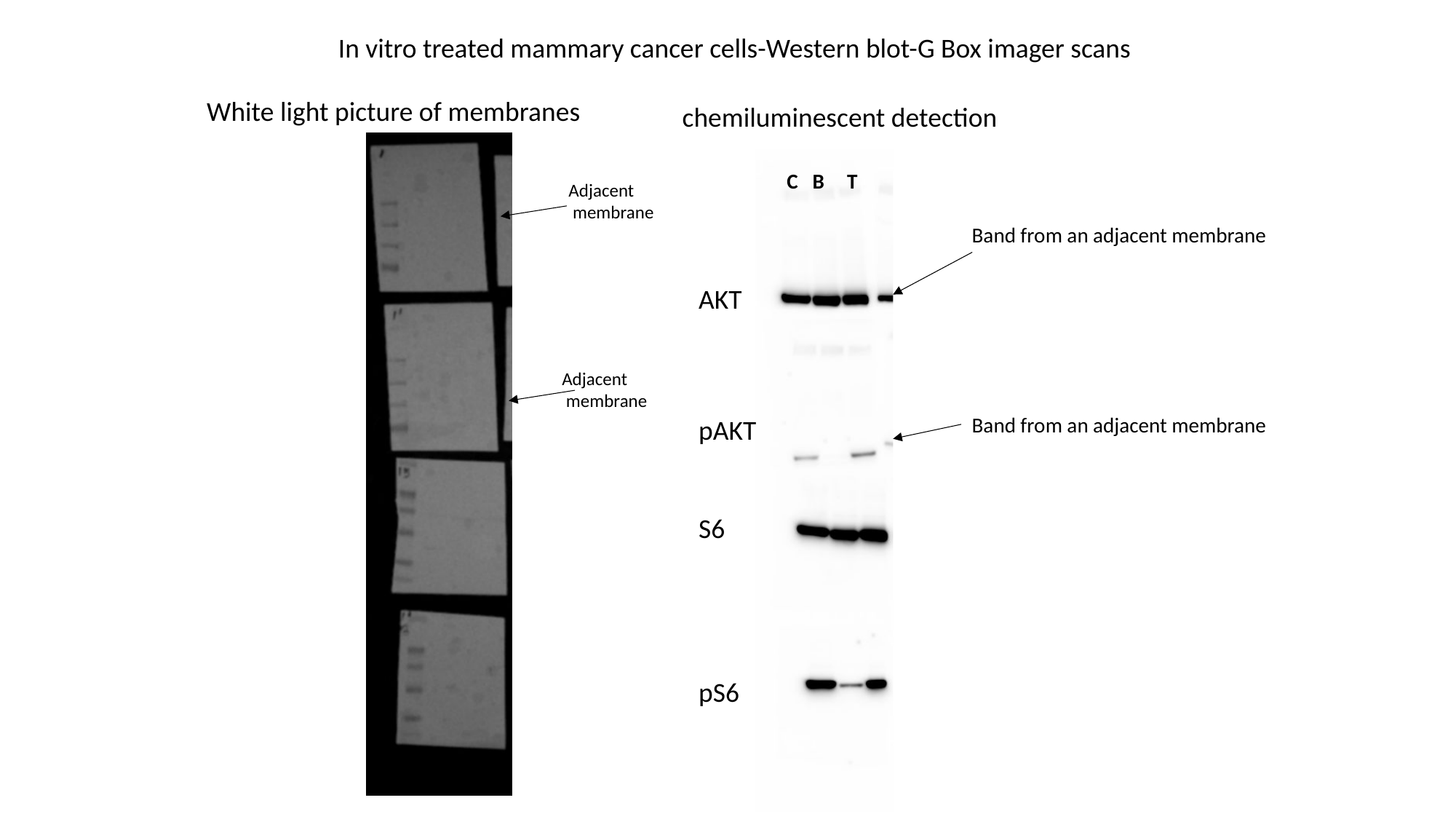

In vitro treated mammary cancer cells-Western blot-G Box imager scans
White light picture of membranes
chemiluminescent detection
C
B
T
Adjacent
 membrane
Band from an adjacent membrane
AKT
pAKT
S6
pS6
Adjacent
 membrane
Band from an adjacent membrane

## Slide 7
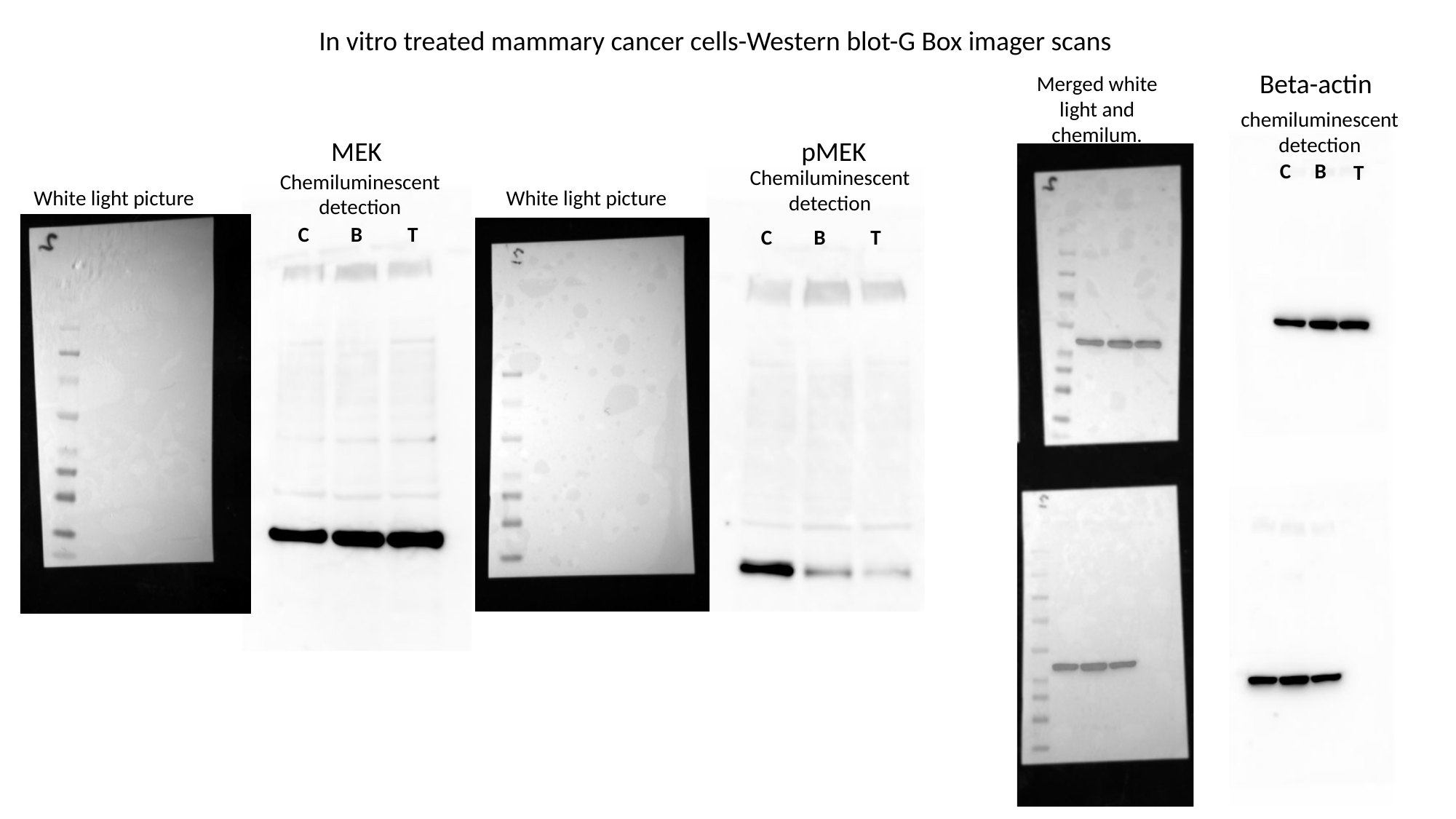

In vitro treated mammary cancer cells-Western blot-G Box imager scans
Beta-actin
Merged white light and chemilum.
chemiluminescent
detection
MEK
pMEK
C
B
T
Chemiluminescent
 detection
Chemiluminescent
 detection
White light picture
White light picture
C
B
T
C
B
T

## Slide 8
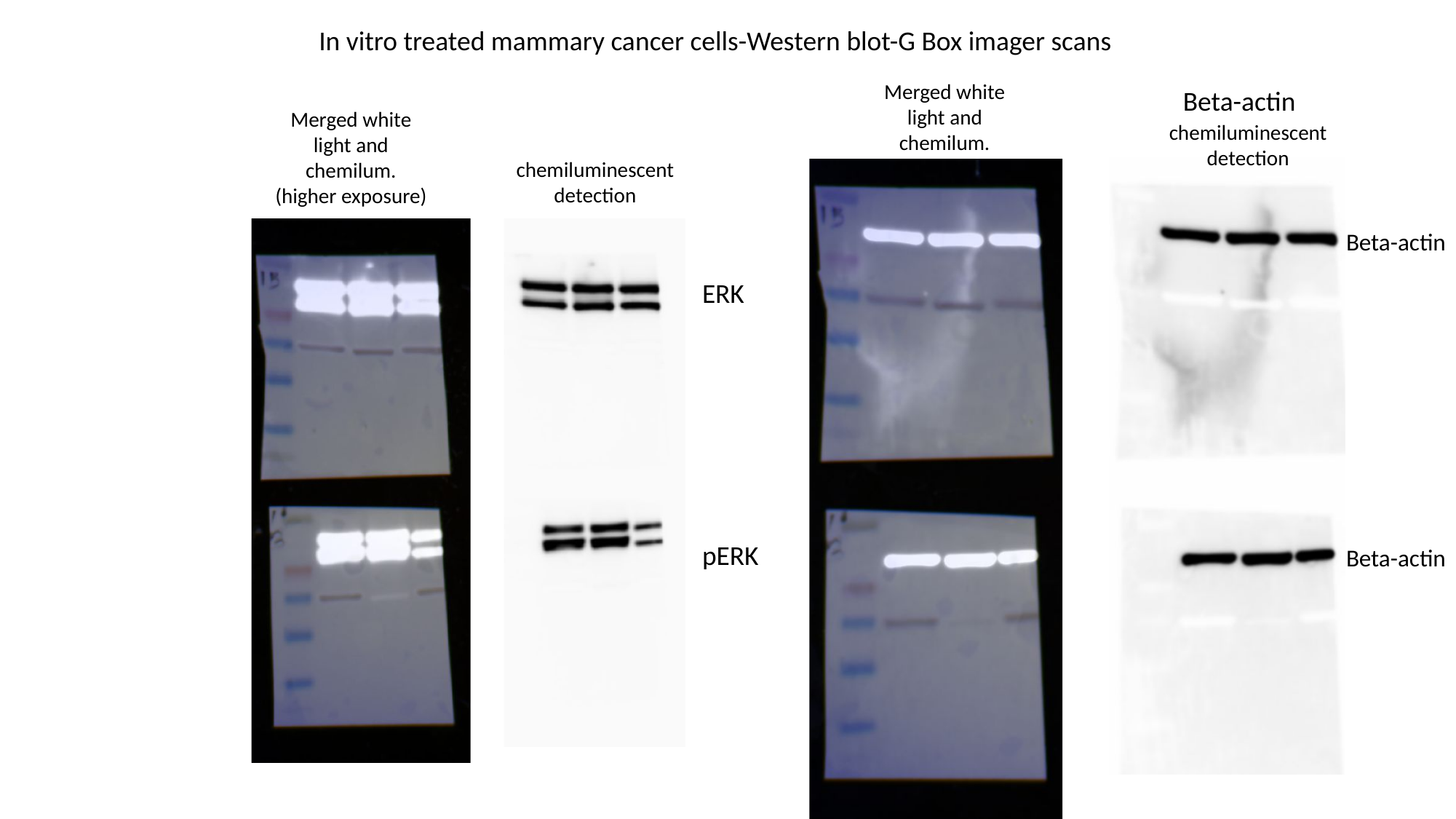

In vitro treated mammary cancer cells-Western blot-G Box imager scans
Merged white light and chemilum.
Beta-actin
Merged white light and chemilum. (higher exposure)
chemiluminescent
detection
chemiluminescent
detection
Beta-actin
ERK
pERK
Beta-actin
