# Supplementary material for: Loss of Brca1 and Trp53 in adult mouse mammary ductal epithelium results in development of hormone receptor-positive or hormone receptor-negative tumors, depending on inactivation of Rb family proteins
Source: Breast Cancer Res. 2022 Nov 4;24:75. doi: 10.1186/s13058-022-01566-4 (PMC9636824; doi:10.1186/s13058-022-01566-4)
Supplement: Supplementary file 17 — Additional file 17. Supplementary Methods. [file 13058_2022_1566_MOESM17_ESM.docx]

**Supplementary Methods**

Quantitative PCR

Quantitative PCR was used to assess the expression of ER and PR in mammary tumors. Total RNA was extracted from mammary tumors using standard protocols and residual DNA was eliminated by DNA-free DNase Treatment & Removal (Ambion by Life Technologies, Carlsbad, CA, USA). One microgram of RNA was used in the synthesis of cDNA by High-Capacity RNA-to-cDNA Kit (Applied Biosystems by ThermoFisher Scientific, Vilnius, Lithuania). cDNA was diluted 1:50 and 8µl of diluted cDNA was subsequently used in qPCR reaction using FastStart Universal SybrGreen Master Mix (Roche, Mannheim, Germany) and 500nM primers. Primer sequences used in qPCR were: ER: 5’- CCAGCAGGTGCCCTACTACC-3’ and 5’- TAGTCATTGCACACGGCACA-3’, PR: 5’-GGTGGGCCTTCCTAACGAG-3’ and 5’-GACCACATCAGGCTCAATGCT-3’, Rb1: 5’- CAGATGGAAGATGACCTGGTA-3’ and 5’- CGCTCCTGTTCTGACCTCTT-3’, P107: 5’- TTCGTGAGCGGATAGAAAGG -3’and 5’- ATACGGAAATTCCCCTTGGT-3’, Cyclin D1: 5’-5’- AACTACCTGGACCGCTTCCT -3’ and 5’- CCACTTGAGCTTGTTCACCA-3’, Cyclin E1: 5’- CTTTCATCCCCACCCCTAAC -3’ and 5’- AGGATGACGCTGCAGAAAGT-3’, CDK2: 5’- AAATTCATGGATGCCTCTGC -3’ and 5’- ACAGGGACTCCAAAGGCTCT-3’, CDK4: 5’- GGGACATCAAGGTCACCCTA -3’ and 5’- CAGCTTGACGGTCCCATTAC-3’, beta-actin: 5’-AGCCATGTACGTAGCCATCC-3’ and 5’-TTTGATGTCACGCACGATTT-3’. PCR was performed on Applied Biosystems 7500 Fast Real-Time PCR System machine using following protocol: initial denaturation at 95°C for 10 min, followed by 40 cycles of denaturation at 95°C for 15 secs, annealing and extension at 60°C for 1 min. The amplification was followed by the melting curve measurement: rapid heating of amplified samples to 95°C to denature the DNA; cooling to 60°C to let DNA strands to anneal; then slowly heating the samples to 95°C, where the change in fluorescence was measured at each 0.1°C rise for 1 second. Amplification of endogenous control, beta-actin, was performed for each sample. All samples were run in triplicates. Comparative C_t_ method was used to evaluate the relative quantity of the target genes using 2^-deltaCt^ method, where delta Ct= mean Ct_target gene_ -Ct_actin_.

PCR to detect recombined alleles

To detect recombination and excision of loxP flanked sequences in *Brca1fl/fl*; *p53fl/fl*; and *K18-T121tg/+* tumors arising from Adeno-Cre injected mammary glands genomic DNA was extracted from bulk tumors using DNeasy Blood & Tissue Kit (Qiagen, Germantown, MD, USA). Primers and primer sequences to detect Cre recombinase-mediated excision of LoxP flanked sequences in were: Brca1 fw: 5’- TATCACCACTGAATCTCTACCG-3’ and Rev: 5’-TCCATAGCATCTCCT TCTAAAC-3’, Trp53 Fw: 5’- CACAAAAACAGGTTAAACCCAG-3’ and Rev: 5’- GAAGACAGAAAAGGGGAGGG-3’ and K18-T121 Fw: 5’-CGTGTGCAGAAGTGGAGAAG-3’ and Rev: 5’-GCATCCCAGAAGCCTCCAAAG-3’. The cycling profile was 95°C for 15 min., 30 cycles of 95°C for 15 sec., 55°C for 30 sec., and 72°C for 30 sec.; the final incubation of 72°C was for 7 min.  PCR products for recombined alleles were resolved on 1% agarose gel in TAE buffer.

Brca1 status in mammary tumors

Brca1 status in tumors was assessed by RT-qPCR. Total RNA was extracted from mammary tumors using standard protocols and residual DNA was eliminated by DNA-free DNase Treatment & Removal (Ambion by Life Technologies, Carlsbad, CA, USA). One microgram of RNA was used in the synthesis of cDNA by High-Capacity RNA-to-cDNA Kit (Applied Biosystems by ThermoFisher Scientific, Vilnius, Lithuania). cDNA was diluted 1:50 and 8µl of diluted cDNA was subsequently used in qPCR reaction using FastStart Universal SybrGreen Master Mix (Roche, Mannheim, Germany) and 500nM primers. Primer sequences used in qPCR were: Brca1: 5’-GGAAGGGTAGCAGCAGTGAC-3’ and 5’-GGGAGTTTGCATTTGCAGTT-3’ and beta-actin: 5’-AGCCATGTACGTAGCCATCC-3’ and 5’-TTTGATGTCACGCACGATTT-3’. PCR was performed on Applied Biosystems 7500 Fast Real-Time PCR System machine using following protocol: initial denaturation at 95°C for 10 min, followed by 40 cycles of denaturation at 95°C for 15 secs, annealing and extension at 60°C for 1 min. The amplification was followed by the melting curve measurement: rapid heating of amplified samples to 95°C to denature the DNA; cooling to 60°C to let DNA strands to anneal; then slowly heating the samples to 95°C, where the change in fluorescence was measured at each 0.1°C rise for 1 second. Amplification of endogenous control, beta-actin, was performed for each sample. All samples were run in triplicates. Comparative C_t_ method was used to evaluate the relative quantity of the target genes using 2^-deltaCt^ method, where delta Ct= mean Ct_target gene_ -Ct_actin_. Expression in B1/P and B1/P/Rb_f_ tumors was normalized to the expression in mammary tumor with wild type Brca1.

Western blot

Mammary cancer cells at 80% confluency were treated with 200nM GSK1120212 (Trametinib (ChemieTek, Indianapolis, IN, USA) or BEZ235 (ChemieTek, Indianapolis, IN, USA) and harvested 4 hrs later for protein preparation. Protein lysates from cells or tumor tissue were prepared using lysis buffer (50mM Tris, pH 7.9, 150mM NaCl, 1mM EDTA, 1% NP40 and 10% glycerol) supplemented with Phosphatase Inhibitor Cocktail 2 and 3 (Sigma, Saint Louis, MO, USA), 1mM Na_3_VO_4_, Complete Mini Protease Inhibitor Cocktail tablets (Roche, Mannheim, Germany), 1mM DTT (Sigma, Saint Louis, MO, USA) and 1mM PMSF (Sigma, Saint Louis, MO, USA).

Western blots were performed by standard methods using the following antibodies: anti-ERK1/2 (p44/42 MAPK, #9101, Cell Signaling Technology, Beverly, MA, USA), anti-phospho-ERK (p44/42 MAPK, #9102, Cell Signaling Technology, Beverly, MA, USA), anti-AKT (clone C67E7, #4691, Cell Signaling Technology, Beverly, MA, USA), anti-phospho-AKT (#4058, Cell Signaling Technology, Beverly, MA, USA), anti-MEK (#9126, Cell Signaling Technology, Beverly, MA, USA), anti-pMEK1/2 (#9154, Cell Signaling Technology, Beverly, MA, USA), anti-S6 (#2215, Cell Signaling Technology, Beverly, MA, USA), anti-pS6 (#2217, Cell Signaling Technology, Beverly, MA, USA), anti-beta actin (#A5441, Sigma, Saint Louis, MO, USA), anti-rabbit IgG-HRP conjugated (#7074, Cell Signaling Technology, Beverly, MA, USA) and anti-mouse IgG HRP conjugated (#GENA931, Sigma, Saint Louis, MO, USA). ECL (#GERPN2209. Sigma, Saint Louis, MO, USA) was used to produce chemiluminescent signal that was detected on film or by G Box Chemi-XRQ imager (Syngene, Frederick, MD, USA).

Fluorescent in situ hybridization (FISH)

Chromosome preparations were obtained from established primary mouse mammary cancer cell lines. Slides were prepared and incubated overnight for use in FISH analysis of Her2 gene. For the detection of the *Her2* gene (*Erbb2*), a full length ORF clone for Mouse *Erbb2* (Sino Biological, Wayne, PA, USA) was obtained and labeled with ATTO 488-dUTP(green) by nick translation. Co-hybridization of *Erbb2* with WCP14, labeled with Orange 552 dUTP, (XMP, MetaSystems, Newton, MA, USA) was used for verification of the location of the cDNA clone. Hybridization was carried out in a humidity chamber at 37°C for 16 hours according to standard protocols. The post-hybridization rapid wash procedure was used with 0.4 x SSC at 72°C for 4 min. Detection was carried out following the manufacturer’s protocol. Spectral images of the hybridized metaphases analyzed using HiFISH 7.2 acquisition software (Genasis, Applied Spectral Imaging, CA). G-banding was simulated by electronic inversion of DAPI counterstaining. Twenty cells were scored.
